# Setting Up Decision-Making Tools toward a Quality-Oriented Participatory Maize Breeding Program

**DOI:** 10.3389/fpls.2017.02203

**Published:** 2017-12-22

**Authors:** Mara L. Alves, Cláudia Brites, Manuel Paulo, Bruna Carbas, Maria Belo, Pedro M. R. Mendes-Moreira, Carla Brites, Maria do Rosário Bronze, Jerko Gunjača, Zlatko Šatović, Maria C. Vaz Patto

**Affiliations:** ^1^Instituto de Tecnologia Química e Biológica António Xavier, Universidade Nova de Lisboa, Oeiras, Portugal; ^2^Instituto Politécnico de Coimbra, Escola Superior Agrária, Coimbra, Portugal; ^3^Unidade de Tecnologia e Inovação, Instituto Nacional de Investigação Agrária e Veterinária, Oeiras, Portugal; ^4^Faculdade de Farmácia, Universidade de Lisboa, Lisboa, Portugal; ^5^Food and Health Division, Instituto de Biologia Experimental e Tecnológica, Oeiras, Portugal; ^6^Faculty of Agriculture, University of Zagreb, Zagreb, Croatia; ^7^Centre of Excellence for Biodiversity and Molecular Plant Breeding (CoE CroP-BioDiv), Zagreb, Croatia

**Keywords:** *Zea mays* L., open-pollinated varieties, yield, nutritional quality, organoleptic quality, processing quality, genetic diversity, participatory plant breeding

## Abstract

Previous studies have reported promising differences in the quality of kernels from farmers' maize populations collected in a Portuguese region known to produce maize-based bread. However, several limitations have been identified in the previous characterizations of those populations, such as a limited set of quality traits accessed and a missing accurate agronomic performance evaluation. The objectives of this study were to perform a more detailed quality characterization of Portuguese farmers' maize populations; to estimate their agronomic performance in a broader range of environments; and to integrate quality, agronomic, and molecular data in the setting up of decision-making tools for the establishment of a quality-oriented participatory maize breeding program. Sixteen farmers' maize populations, together with 10 other maize populations chosen for comparison purposes, were multiplied in a common-garden experiment for quality evaluation. Flour obtained from each population was used to study kernel composition (protein, fat, fiber), flour's pasting behavior, and bioactive compound levels (carotenoids, tocopherols, phenolic compounds). These maize populations were evaluated for grain yield and ear weight in nine locations across Portugal; the populations' adaptability and stability were evaluated using additive main effects and multiplication interaction (AMMI) model analysis. The phenotypic characterization of each population was complemented with a molecular characterization, in which 30 individuals per population were genotyped with 20 microsatellites. Almost all farmers' populations were clustered into the same quality-group characterized by high levels of protein and fiber, low levels of carotenoids, volatile aldehydes, α- and δ-tocopherols, and breakdown viscosity. Within this quality-group, variability on particular quality traits (color and some bioactive compounds) could still be found. Regarding the agronomic performance, farmers' maize populations had low, but considerably stable, grain yields across the tested environments. As for their genetic diversity, each farmers' population was genetically heterogeneous; nonetheless, all farmers' populations were distinct from each other's. In conclusion, and taking into consideration different quality improvement objectives, the integration of the data generated within this study allowed the outline and exploration of alternative directions for future breeding activities. As a consequence, more informed choices will optimize the use of the resources available and improve the efficiency of participatory breeding activities.

## Introduction

Maize (*Zea mays* L.) plays a major role in nutrition in many countries, and is the basis for the production of several foods, such as polenta, bread, tortillas, snacks, and cornflakes (Fernandes et al., 2013). In some of countries such in Spain or Portugal whole maize flour is used for bread production (Rodríguez et al., 2013). The ethnic Portuguese maize-based bread is known locally as *broa*. *Broa* is traditionally made with more than 50% maize flour mixed with rye and/or wheat flour in a mostly empirical process (Brites et al., 2010). As further described by the same authors (Brites et al., 2010), this process normally involves the mixing of sieved wholemeal maize flour with hot water, rye, and/or wheat flour (in a variable proportion), with yeast from leavened dough from earlier *broa* acting as sourdough.

In the last few decades, consumers' views on how foods positively or negatively affect their health have changed and, therefore, foods today are not only intended to satisfy hunger and provide necessary nutrients; they are also used to prevent nutrition-related diseases and improve physical and mental well-being (reviewed in Siró et al., 2008). Given this rising awareness in consumers, the consideration of the quality aspects of plant breeding is now a commercially relevant issue. The health benefits of consuming whole grains have been well documented, and are often associated with those benefits conveyed by their dietary fiber content (Ktenioudaki et al., 2015). Additionally, whole grains are rich in bioactive phytochemicals such as phenolic compounds, tocopherols, and carotenoids (Slavin et al., 2000).

Additionally, the market demand for gluten-free formulations has driven more research in the different steps leading from the maize kernel to the maize bread quality (e.g., Moreira et al., 2015; Garzón et al., 2017; Martínez and Gómez, 2017). In parallel, an increased investment on the improvement of open-pollinated maize populations has been driven by a renewed interest in materials traditionally used for ethnic food commodities and for their use in the context of more sustainable farming systems (e.g., Revilla et al., 2012, 2015; Samayoa et al., 2016).

Since the introduction of maize in Europe from the Americas in the fifteenth century, diverse maize varieties have been selected for adaptation to a wide range of environments and consumer preferences (Tenaillon and Charcosset, 2011; Revilla et al., 2015). Portugal, Spain, and Italy are considered primary centers of maize introduction in Europe (Dubreuil et al., 2006). The European maize populations although much less variable than the Central and South American populations (Rebourg et al., 2003), are a useful alternative because they were selected from multiple origins in the Americas and have the advantage of 400 years of adaptation to temperate climates (Romay et al., 2012), but lower yield than modern hybrids under conventional agricultural conditions (Revilla et al., 2015).

In the twenty-first century, Portuguese traditional maize populations can be still found under production as verified in a collecting expedition that took place in the last decade in the Northern Central region of Portugal (Vaz Patto et al., 2007). This mission had as its main objective sampling the enduring traditional maize populations' variability in a particular region of the country, where maize-based bread still plays an important role in the local rural economy (Vaz Patto et al., 2007). In this collecting expedition it was recorded that the majority of the maize populations conserved were being used primarily for bread production. As a consequence, the collected populations were assumed to have the potential to be used in *broa* production. The fact that flour produced from locally grown maize populations has traditionally been used in the formulation of *broa* has been pointed out by Vaz Patto et al. (2007) as one of the reasons for the on-farm conservation of the Portuguese maize populations. Brites et al. (2010), through a sensory analysis on *broa* bread carried out by a trained panel using open-pollinated maize populations, identified a preference, due to texture, taste, and aroma, for maize bread produced using open-pollinated populations, as opposed to maize bread produced using commercial hybrid maize varieties. In the same study, instrumental quality attributes of maize flour from open-pollinated populations were measured and compared to commercial hybrid maize varieties. The results from that study showed that the flour from open-pollinated populations—considered by the trained panel to produce better quality *broa*—had higher values of protein, lower values of amylose, and lower viscosities (maximum, minimum, final, and breakdown viscosities) (Brites et al., 2010).

Besides the phenotypic characterization, a better understanding of the genetic diversity present in the germplasm available for breeding helps to structure germplasm, defining, for example, heterotic pools; provides useful information for selecting contrasting parental lines for new breeding populations; and helps breeders to identify valuable new alleles for breeding (Varshney et al., 2016).

Currently, only a limited number of Portuguese traditional maize populations are integrated in a long-term participatory maize breeding program that has been running since 1984 in the northeast region of Portugal (Sousa Valley, Lousada; Vaz Patto et al., 2013). One of the main advantages of on-farm participatory plant breeding is that it enables the constant adaptation of crops to the environment and supports the involvement of farmers since the selection criteria for the maize populations are defined in accordance with farmers' decisions. This breeding program was set at the Sousa Valley region because this was a well-known area in the country for maize production, with good edaphic-climatic conditions, and because at the time of the program implementation, it was initiated with the support of the local community (reviewed in Vaz Patto et al., 2013). In the Portuguese participatory maize breeding program, selection was mainly focused on the improvement of grain yield and other important agronomic traits, considering that quality was safeguarded by the use of local traditional maize populations (Moreira, 2006). Nevertheless, by the comparative evaluation of different selection cycles of some of the participatory bred maize populations, Alves et al. (2017) concluded that although diversity was maintained under this program, quality evolved erratically. This observation, together with the increasing market importance given to quality aspects, set the stage for addressing the need to develop appropriate decision-making tools to bring about a quality-oriented maize population selection.

Although previous works (Vaz Patto et al., 2007, 2009; Brites et al., 2010) improved our knowledge of the agronomic, quality, and molecular aspects of traditional maize populations collected from the central region of Portugal, some limitations remained. Specifically, in terms of agronomic characterization, it is still necessary to understand the eventual effect and interaction of the different maize farming sites on those maize populations. Moreover, the use of controlled pollinations in the previous studies might have reduced production per plot, as described in Vaz Patto et al. (2007); therefore, field trials, under real production management over several locations, are still necessary to correctly evaluate the potential grain yield and to study how each traditional population behaves when grown in the different areas where maize populations have traditionally been produced in the country. In terms of quality characterization, it is necessary to evaluate other health-promoting, nutritional, and organoleptic compounds that can have an impact on consumers' perception and acceptance of the final product. Finally, in terms of molecular characterization, it is necessary to increase the number of individual plants evaluated per population from the original five. Maize is a naturally open-pollinated crop and, therefore, a large number of individuals should be evaluated to accurately estimate the number of alleles and their frequency per population and, as a result, to assess the similarities and infer the genetic structure between and within maize populations.

The maize populations that were surveyed in the collecting mission that took place in the Central-Northern region of Portugal (Vaz Patto et al., 2007) are not at this date involved in any participatory maize breeding program. Given the previous Portuguese experience with this type of breeding approach and to promote the use of such distinct material, this work proposes to produce relevant (phenotypic and molecular) information on these materials, and to develop decision-making tools to aid in the establishment of a quality-oriented participatory breeding program. This breeding program should take into consideration market-driven quality traits (traits related to consumer acceptance, such as organoleptic and health-related compounds), while also improving the agronomic performance of the breeding materials. The characterization of these populations will allow the identification of the most relevant ones for each breeding objective and will result in a more efficient use of those genetic resources in breeding programs.

Therefore, the objectives of this study are:

1. To extend the maize populations quality characterization—organoleptic, nutritional, and health-related traits—with the quantification of aroma-related volatile compounds, and health-related compounds, such as tocopherols, carotenoids, and phenolic compounds, that might influence the quality of maize-based food commodities;
2. To accurately estimate the agronomic performance and potential of the collected maize populations using multi-location field trials (broader performance stability/specific adaptability) across different farming sites, exploring new locations for the establishment of a future quality-oriented participatory maize breeding program;
3. To build decision-making tools to enable an accurate population selection within a quality-oriented participatory breeding program, by complementing the precise agronomic and quality description with a more thorough molecular characterization.

## Materials and methods

### Plant material

The materials evaluated in this study consisted of 16 enduring traditional maize populations that were collected in the Central Northern region of the country from small farms with low input agricultural systems (Vaz Patto et al., 2007). These farmers' populations were labeled in this work as *broa-x* (*x* corresponds to the specific name given to each population).

For comparison purposes, nine open-pollinated populations from the long-term Portuguese maize participatory breeding program, identified in this work as participatory bred (PPB) populations, and an international reference, the US open-pollinated population *BS22(R)C6*, were also included in this study. The populations under the Portuguese maize participatory breeding program were selected and/or developed primarily to improve their agronomic performance (reviewed in Vaz Patto et al., 2013). *BS22(R)C6* is a genetically broad-based synthetic population developed primarily for improved grain yield and root and stalk strength (Hallauer et al., 2000). More information about each population can be found in Table S1.

### Quality evaluation

Quality traits related to flour's pasting behavior (flour viscosity parameters), nutritional value (protein, fat, and fiber content), bioactive compounds (carotenoids, tocopherols, total phenolic content, *p*-coumaric, and ferulic acid content), and aroma-related compounds (volatile aldehydes content) were evaluated in 26 maize populations. For that, a bulk of grain from each maize population produced from a common-garden experiment established in Coimbra in 2009 was used. Information about the site characterization can be found in Table S2. Each population was overplanted by hand in two-row plots 6.4 m long and with 0.75 m border space between two planted rows. Each plot was thinned at the seven-leaf stage to 48 plants per plot to achieve a plant density of 50,000 plants.ha^−1^. Plots were irrigated as needed and mechanically and/or hand weeded as necessary following common agricultural practices for maize in the region. Pollination was controlled within each plot. All the plots were harvested by hand. After harvest, ears were dried at 30–35°C in an oven (Memmert Model UFE 800, Memmert GmbH + Co. KG, Germany) until a ~15% in moisture was reached. The ears were then shelled and the kernel collected per plot basis, packed in a paper bags and kept at 4°C until further analysis.

Wholemeal maize flour was obtained after milling the kernel through a Cyclone Falling number 3100 mill (Perten, Sweden) with a 0.8 mm mesh.

The pasting properties of maize flour were obtained with a Rapid Viscosity Analyzer RVA-4 (Newport Scientific, Australia). The viscosity profiles were obtained for each population according to Almeida-Dominguez et al. (1997) at 15% solids, using the following heating and cooling cycle settings: (1) holding at 50°C for 2 min, (2) heating to 95°C in 4.5 min, (3) holding at 95°C for 4.5 min, (4) cooling to 50°C in 4 min, (5) holding at 50°C for 10 min. The RVA paddle speed was set at 960 rpm for the first 10 s of the test, after which the speed was changed to 160 rpm. Peak (PV), minimum or trough (TV), and final viscosities (FV) were recorded in cPoise and the breakdown viscosity (BD) was calculated as PV–TV, and setback from trough viscosity (SB1) was calculated as FV–TV.

Maize flour yellowness was determined on a 10–12 g sample in an opaque recipient using a Minolta chromameter CR-2b and the CIE tristimulus color parameters b^*^ (yellow/blue index). Positive b^*^ values indicate that sample tends toward the yellow part of the color spectra.

Flour protein (PR), fat (FT), and fiber (FI) content were determined by a near-infrared spectroscopic method using Inframatic 8620 equipment (Perten, Sweden), with calibrations supplied by the manufacturer. Results were expressed in percentages.

The total carotenoids content (TCC) was spectrophotometrically measured at 450 nm according to the AACC method 14-60.01 (AACC International, 2012). Results were expressed in micrograms of lutein equivalent per gram of sample, as the main carotenoid found in maize.

α-Tocopherol (AT), γ-tocopherol (GT), δ-tocopherol (DT) were separated from the fat portion of the maize flours by high-performance liquid chromatography (HPLC) and quantified using an Agilent 1200 model with a fluorescence detector (FLD) and a Diol column (LiChropher 100, 250 × 4 mm) according to the method ISO 9936 (2006). Tocopherols content were expressed in μg/g fat basis.

For assessing the total free phenolic compounds content (PH) of maize flour ethanolic extracts (EtOH:H2O 50:50, v/v) were prepared according to Lopez-Martinez et al. (2009), with some modifications. Briefly, 2 g of maize flour was extracted with 20 mL of EtOH:H_2_O (50:50, v/v) for 15 min, using an Ultra Turrax T25 (Janke & Kunkel, IKA Labortechnik, Germany). Final extracts were filtered using a Whatman filter paper (type42: retention 2.5 μm, diameter 18.5 cm). Extracts were prepared in triplicate and preserved at −20°C until analysis.

Total free phenolic compounds content (PH) was assessed using the Folin-Ciocalteau assay (Singleton et al., 1999) with a Beckman DU-70 spectrophotometer, with slight modifications as described in Silva et al. (2015), and expressed in mg of gallic acid equivalents/100 g of dry weight (GAE/100 g DW).

*p*-Coumaric (CU) and ferulic acid (FE) were quantified by HPLC coupled with a photodiode array detector (HPLC-PDA) at 280 nm with a Thermo Finnigan Surveyor HPLC system according to Silva et al. (2006). *p*-Coumaric (CU) and ferulic acid content were expressed in mg/100 g of dry weight.

Solid phase micro-extraction (SPME) was used as sample preparation methodology and the volatile fraction was analyzed by gas chromatography—mass spectrometry (SPME-GC-MS). Briefly, to 1 g of maize flour, 4.5 mL of Milli-Q water was added to a capped vial and were homogenized using a vortex. For sample preparation a 2 cm−50/30 μm DVB/Carboxen/PDMS fiber (SUPELCO) and an exposure time of 60 min, at 60°C were used.

Volatile compounds were analyzed in a GCMS-QP2010 Plus Shimadzu equipment and compound were separated in a Varian Factor Four column (30 m × 0.25 mm × 0.25 μm). The injector was at 250°C and the column was at 35°C for 5 min, followed by a gradual increase of 5°C/min until a final temperature of 230°C was reached. Injection was performed using a splitless mode. The interface and ion source on MS equipment were set at 250°C. Mass spectra were produced at 70 eV in a range of 29–299, using a scanning velocity of 555 scans/s. Helium was used as mobile phase at a flow rate of 2.1 mL/ min. The equipment was coupled to an automatic sampler AOC-5000 (Shimadzu). GCMSsolution Release 2.53SU1 software was applied for data acquisition and treatment.

Volatile aldehydes content (AL) was taken as the sum of the peak area of the main aldehydes identified [hexanal, heptenal, 2-heptanal (*Z*), 2-octenal (*E*), nonanal, 2-nonenal (*E*) and decanal]. Identification of volatile compounds was performed by a comparison of the experimental mass spectra with the ones from the software's spectra library (WILEY 229, NIST 27 and 147). A standard mixture of hydrocarbons C8-C20 (40 mg/L each, in hexane) was used to determine linear retention indexes—LRI (Kovats indexes)—in order to confirm identification. The values of LRI determined for each compound were compared with described LRI for the same type of column (El-Sayed, 2014, http://www.pherobase.com).

### Quality data analysis

All the calculations were performed in SAS Release 9.2 (SAS Institute Inc., 2004). Pearson correlation coefficients were calculated between the 14 maize quality traits in all maize populations using PROC CORR procedure.

Principal component analysis (PCA) was performed using the PROC PRINCOMP procedure on standardized data. The number of principal components was determined by checking eigenvalues of the principal components (Kaiser Criterion that retains components with eigenvalues greater than one and SCREE plot) and the cumulative proportion of variance explained.

The standardized principal component scores were multiplied by the root of their eigenvalues to calculate pairwise Euclidean distances between populations. The average linkage method (i.e., UPGMA) of PROC CLUSTER was applied in order to classify maize populations into groups and to determine the optimal number of clusters. Cubic Clustering Criterion (CCC) statistics and Pseudo F (PSF) statistics were calculated and plotted. The classification of maize populations into groups as obtained by cluster analysis was evaluated by discriminant analysis (DA) using 14 traits in PROC DISCRIM procedure in SAS. The probabilities of classification success of the discriminant function were estimated by cross-validation.

The univariate analysis of variance using PROC GLM was conducted in order to test mean differences between quality-groups for 14 traits. Means were separated using the least-squares means procedure with Tukey's control adjustment for multiple comparisons.

### Agronomic evaluation

The agronomic performance of all maize populations was compared in multi-location field trials. Field trials were established during 2010 in nine different sites: Quinta da Conraria, Montemor-o-Velho, S. Pedro do Sul, Lousada, Valada do Ribatejo, Vouzela-1, Vouzela-2, Travassos, and Coimbra.

The different locations represent different areas where maize open-pollinated populations traditionally are produced in the country and the different agronomic production systems normally associated with maize open-pollinated populations, ranging from conventional (Montemor-o-Velho) to organic (Quinta da Conraria and Valada do Ribatejo), and also considering low-input production systems (all the other locations). Information about the sites' characterizations can be found in Table S2.

During the 2010 growing season, a total of 26 maize populations were evaluated in a randomized complete block design, each population replicated within the three blocks set per field trial (location). Each population was overplanted by hand in two-row plots 6.4 m long and with 0.75 m between rows. Each plot was thinned at the seven-leaf stage to 48 plants per plot to achieve a plant density of 50,000 plants.ha^−1^. Plots were irrigated as needed and mechanically and/or hand weeded as necessary. All the plots were harvested by hand.

In each environment, a maximum of 144 plants (48 plants per plot × 3 blocks) were evaluated for each population. Missing data issues were identified for all the late cycle populations (*Verdeal da Aperrela, Castro Verde, Estica, Fisga*, and *Fandango*) in Travassos, Vouzela-1, and S. Pedro do Sul; all sites located at mid altitude, where no data was obtained. The *Pigarro* population, a participatory bred population, also suffered from poor adaptation to the trial environments since data for *Pigarro* could only be retrieved for three out of nine environments: Lousada (the population's site of origin), Valada do Ribatejo, and Vouzela-2, the latter with data in only one block.

Grain yield and ear weight per population were recorded for each block. Ear weight was taken as an indirect measurement of ear size, the trait for which the majority of the collected maize populations were being selected. The agronomic performance of each population was evaluated according to Moreira et al. (2008) as described in Table S3.

### Agronomic data analysis

Pearson correlation coefficients between grain yield and ear weight were calculated using PROC CORR procedure in SAS Release 9.2 (SAS Institute Inc., 2004). Given the high correlation between grain yield and ear weight further analysis on genotype by environment interactions was reported for grain yield only.

The genotype-by-environment (G × E) interaction analysis was carried out using Additive Main effects and Multiplication Interaction (AMMI) models, a convenient tool for detecting patterns and systemic trends that can usually have direct ecological or biological interpretation (Gauch et al., 2011). Previously described missing data issues required the model fitting using the Expectation-Maximization (EM) algorithm, as implemented in the so-called “EM-AMMI” model (Gauch and Zobel, 1990).

The general form of AMMI models can be expressed as (Gauch, 1992):

$$\begin{array}{l} Y_{ij}=\;\mu +g_{i}+e_{j}+\;\overset{p}{\underset{k=1}{\sum }}\lambda_{k}\gamma_{ik}\delta_{jk}+\;\rho_{ij}+\;\varepsilon_{ij} \end{array}$$

where *Y*_*ij*_ is the mean response of the population *i* in the environment *j*; μ is the overall mean; g_*i*_ is the fixed effect of the population *i* (*i* = 1, 2, … g); *e*_*j*_ is the fixed effect of environment *j* (*j* = 1, 2, … e); ε_*ij*_ is the experimental error; the G × E interaction is represented by the factors λ_*k*_, a singular value of the *k*th interaction principal component axis (IPCA) (*k* = 1, 2, … p, where p is the number of axes to be retained in the model), γ_ik_, the population eigenvector for *k*th IPCA, and δ_*jk*_, the environmental eigenvector for *k*th IPCA; ρ_*ij*_ is the residual comprised of the discarded axes.

Selection of the optimal model (number of axes to be retained in the model) was done by cross-validation, using two replicates for model fitting and the remaining one for validation in 1,000 iterations. Both EM-AMMI modeling and cross-validation were carried out using MATMODEL software (Gauch, 2007).

After selecting the optimal AMMI model, the adaptability and phenotypic stability of the maize populations were summarized in a biplot. Since the optimal model was AMMI1, the biplot depicts the main effects of population/genotype and environment vs. the scores for first IPCA. The biplot was generated in Microsoft Excel 2010 using the IPCA scores and trait means retrieved from MATMODEL software.

### Molecular evaluation

Thirty random individual plants from each maize population were genotyped with 20 microsatellites (SSRs—simple sequence repeats). SSRs were chosen based on their location in the maize reference genome (1 SSR per chromosome arm), and repeat motifs (≥ 3 base pairs) to facilitate allele scoring (Table S4). Information about each SSR can be found at MaizeGDB (Lawrence et al., 2008—www.maizegdb.org).

Genomic DNA was isolated from the adult leaves of each plant using the modified CTAB procedure as described in Saghai-Maroof et al. (1984). Genotyping procedures were carried out accordingly to Alves et al. (2017). A genotypic matrix of the alleles' scores per individual plant, in base pairs, was generated and served as the basis for the molecular data analysis.

### Molecular data analysis

The informativeness of each microsatellite marker was assessed measuring their Polymorphism Information Content (PIC; Botstein et al., 1980) and the number of alleles detected using PowerMarker software (PowerMarker V3.23, Liu and Muse, 2005).

Genetic variability within each population was accessed by the following parameters: the average number of alleles per locus (N_av_), the number of private alleles (N_pr_), using GENEPOP software (GENEPOP V4.0, Raymond and Rousset, 1995), and the allelic richness (N_ar_), as the measure of the number of alleles per locus independent of sample size, using FSTAT software (FSTAT V2.9.3.2, Goudet, 2002).

Also for each population the following parameters based on the allelic frequencies were estimated: the observed (H_O_) and expected heterozygosity (H_E_), and the inbreeding coefficient (F_IS_), using GENEPOP software (GENEPOP V4.0, Raymond and Rousset, 1995). The same software was also used to test if the genotypic frequencies in each population were in conformance to Hardy-Weinberg (HW) expectations. The probability test for Hardy-Weinberg (HW) equilibrium was based on the Markov chain method (Guo and Thompson, 1992; Raymond and Rousset, 1995) followed by sequential Bonferroni adjustments (Rice, 1989) to correct for the effect of multiple tests, using SAS Release 9.2 (SAS Institute Inc., 2004).

For comparison purposes, the significance of differences in average values of N_ar_, H_O_, H_E_, and F_IS_ between farmers' populations and participatory bred (PPB) populations were tested using FSTAT software (FSTAT V2.9.3.2, Goudet, 2002).

The genetic differentiation between all pairs of populations was measured with pairwise F_ST_ estimates. Pairwise F_ST_ values and their respective *P*-values for significant differences from zero were calculated with FSTAT software (FSTAT V2.9.3.2, Goudet, 2002).

To represent the genetic relationships between all maize populations, pairwise Cavalli-Sforza–Edwards' chord distances (D_CSE_) (Cavalli-Sforza and Edwards, 1967) were calculated and an unrooted phylogenetic tree was constructed using Fitch-Margoliash algorithm (Fitch and Margoliash, 1967) with 1,000 bootstraps (Felsenstein, 1985) over microsatellite loci as implemented in SEQBOOT, GENDIST, FITCH, and CONSENSE programs of the PHYLIP software package (PHYLIP ver3.6b, Felsenstein, 2004).

The analysis of molecular variance (AMOVA, Excoffier et al., 1992) was used to partition the total microsatellite diversity among all populations and within all populations. The same analysis was also used to partition the total microsatellite diversity detected among farmers' PPB populations, within farmers' populations vs. participatory bred populations, and within all populations. The variance components retrieved from AMOVA analysis were used to calculate a series of statistics called ϕ-statistics, which summarize the degree of differentiation between population divisions and are analogous to Wright's F-statistics (Excoffier et al., 1992). The variance components were tested statistically by non-parametric randomization tests using 10,000 permutations in ARLEQUIN software (ARLEQUIN ver3.0, Excoffier et al., 2005).

A model-based clustering method was applied on multilocus microsatellite data to infer genetic structure and define the number of gene pools in the dataset using the STRUCTURE software (STRUCTURE V2.3.3, Pritchard et al., 2000). Given a value for the number of gene pools, this method assigns individual genotypes from the entire sample to gene pools in a way that linkage disequilibrium (LD) is maximally explained. Ten runs per each K were done by setting the number of gene pools (K) from 1 to 10. Each run consisted of a burn-in period of 200,000 steps followed by 10^6^ MCMC (Monte Carlo Markov Chain) replicates assuming an admixture model and correlated allele frequencies. No prior information was used to define the gene pools. The choice of the most likely number of gene pools (K) was carried out by comparing the average estimates of the likelihood of the data, ln[Pr(X|K)], for each value of K (Pritchard et al., 2000), as well as by calculating an ad hoc statistic ΔK, based on the rate of change in the log probability of data between successive K values as described by Evanno et al. (2005). The program STRUCTURE HARVESTER was used to process the STRUCTURE results files (STRUCTURE HARVESTER v0.6.92, Earl, 2012).

## Results

### Quality evaluation

Correlations among quality traits can be found in Table S5. The majority (~70%) of the quality traits were not correlated with each other, or had weaker correlations (46.34% of the total significant correlations detected), with a Pearson correlation coefficient |r| < 0.5. Protein (PR) content that was strongly positively correlated with fiber (FI) content (*r* = 0.954, *P* < 0.001). In addition, both these traits (PR and FI) were negatively correlated with the breakdown viscosity (BD) (*r* = −0.752 and *r* = −0.711, respectively, *P* < 0.001), and with the α-tocopherol (*r* = −0.764 and *r* = −0.786, respectively, *P* < 0.001) and δ-tocopherol values (*r* = −0.693 and *r* = 0.719, respectively, *P* < 0.001). The TCC was strongly positively correlated with the flour yellowness (*r* = 0.985, *P* < 0.001), measure as b^*^ from the CIE tristimulus color parameters.

Because the parameters describing the pasting properties of maize flour were correlated among them, and because the breakdown viscosity (BD) and setback from trough viscosity (SB1) parameters were derived from the primary viscosity parameters (FV, PV, and TV), only the BD and SB1 viscosity parameters were chosen for further analyses.

A PCA on the standardized quality data was performed in order to summarize multivariate similarities among the maize populations analyzed.

**Figure 1:**
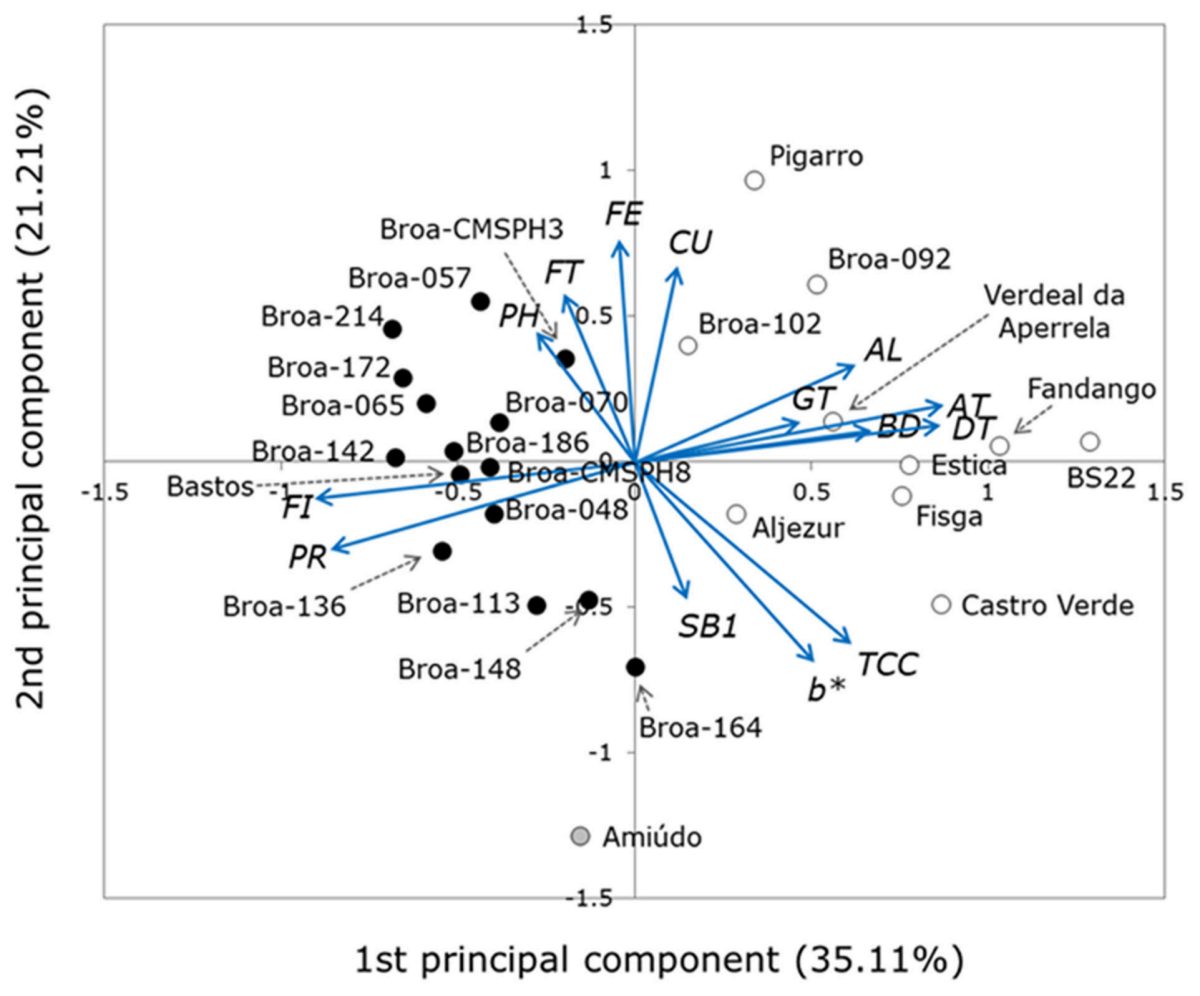
Biplot of principal component analysis (PCA) based on 14 quality traits measured in 26 maize populations; different colored circles correspond to the different quality-based groups identified on cluster analysis: quality-group I is depicted in black, quality-group II is depicted in white; Amiúdo population is depicted in gray.

The position of the maize populations along the first principal component (*x* axis) in the PCA biplot, as shown in Figure 1, was mainly defined by their protein and fiber content, the breakdown viscosity, the TCC, α- and δ-tocopherol content, and volatile aldehydes content. As shown in Figure 1, the farmers' populations (*broa-x* populations) were largely discriminated from the non-*broa-x* maize populations along this principal component. The position of the maize populations along the second principal component (*y* axis) was set primarily according to its flour yellowness (measured by b^*^ color parameter), TCC, *p*-coumaric acid, and ferulic acid content. The third principal component was mainly influenced by setback from trough viscosity values, and the fourth principal component was mainly defined by the levels of total free phenolic compounds (Table S6).

To assess if the different maize populations under study would group into different quality-based groups, a cluster analysis was performed based on the first four principal components retrieved from the PCA. The first four principal components were used since we observed that only by considering the first four principal components, retrieved in the PCA, was a stabilized accumulated percentage of variance (77.94% of total variance) obtained, all having eigenvalues greater than one (Table S6).

As a result of the cluster analysis, the highest values of both Pseudo F (PSF) statistics and CCC were obtained when considering three clusters. Therefore, it was decided that the classification of maize populations in three quality-groups would be the optimal solution. One of the clusters is composed exclusively of one population, the *Amiúdo* population, and was therefore excluded from further analyses. As for the other two quality-groups identified, one was mainly composed of farmers' populations (*broa-x* populations), and was named quality-group I; the second group identified was composed of the remaining maize populations, and was named quality-group II (Figure 1).

The groups retrieved from cluster analysis were then validated by performing a discriminant analysis. The discriminant function, based on 14 traits, correctly classified all the populations into their respective quality-group (100% classification success) when using the standard method, and 22 out of 25 populations (88% classification success) when using the cross-validation method. The groups obtained by cluster analysis were in agreement with the populations' positions in the PCA biplot (Figure 1).

Quality-group I, where the majority of farmers' populations were clustered, was characterized by having a higher fiber and protein content than the average value found in quality-group II, and lower breakdown viscosity values, lower TCC, lower levels of volatile aldehydes, and lower α-tocopherol and δ-tocopherol content than the average values found in quality-group II (Table 1).

**Table 1:** Analysis of variance and comparison of mean values for the quality traits among quality-group I and quality-group II, as defined by cluster analysis.

**No**.	**Trait**	**Mean square**	**P(F)^a^**	**Quality-group**	
				**I**	**II**
1	Protein (PR)	31.89	^***^	12.18	9.83
2	Fiber (FI)	0.87	^***^	2.36	1.97
3	Fat (FT)	1.47 × 10^−5^	ns	4.97	4.97
4	Breakdown (BD)	2,537,542.80	^***^	82.38	746.11
5	Setback1 (SB1)	933,091.60	ns	1,971.63	2,374.11
6	Yellow/blue index (b*)	211.46	ns	16.72	22.78
7	Total carotenoids (TCC)	2,307.99	^*^	15.86	35.88
8	α-tocopherol (AT)	20,068.17	^***^	39.29	98.32
9	δ-tocopherol (DT)	627.43	^***^	16.21	26.65
10	γ-tocopherol (GT)	8,490.42	ns	244.26	282.65
11	Total free phenolic compounds (PH)	1,083.35	ns	159.64	145.92
12	*p*-coumaric acid (CU)	5.48 × 10^−3^	ns	0.35	0.38
13	Ferulic acid (FE)	4.48 × 10^−4^	ns	0.38	0.38
14	Volatile aldehydes (AL)	6.84 × 10^14^	^***^	2,440,756.40	13,337,032.00

a P(F), Significance of the F-test for differences between quality groups; ns, non-siginificant;

* Significant at P < 0.05;

*** *Significant at P < 0.001*.

Quality traits' units: Protein (PR), fiber (FI) and fat (FT) expressed in percentage; Viscosity parameters (BD and SB1) expressed in cPoise; Yellow/blue index (b^*^)—if b^*^ is positive it means that samples tend to the yellow part of the color spectra; Total carotenoids (TCC) expressed in μg of lutein equivalent per gram of sample; Tocopherols (AT, DT, and GT) expressed in μg/g fat basis; Total free phenolic compounds content (PH) expressed in gallic acid equivalents/100 g of dry weight; p-coumaric acid (CU) and ferulic acid (FE) expressed in mg/100 g of dry weight; Aldehydes (AL) taken as the chromatogram peak area.

### Agronomic evaluation

Grain yield was strongly and positively correlated with ear weight (*r* = 0.81, *P* < 0.0001), therefore the following Genotype-by-Environment interaction analysis on agronomic data was reported only for grain yield.

**Figure 2:**
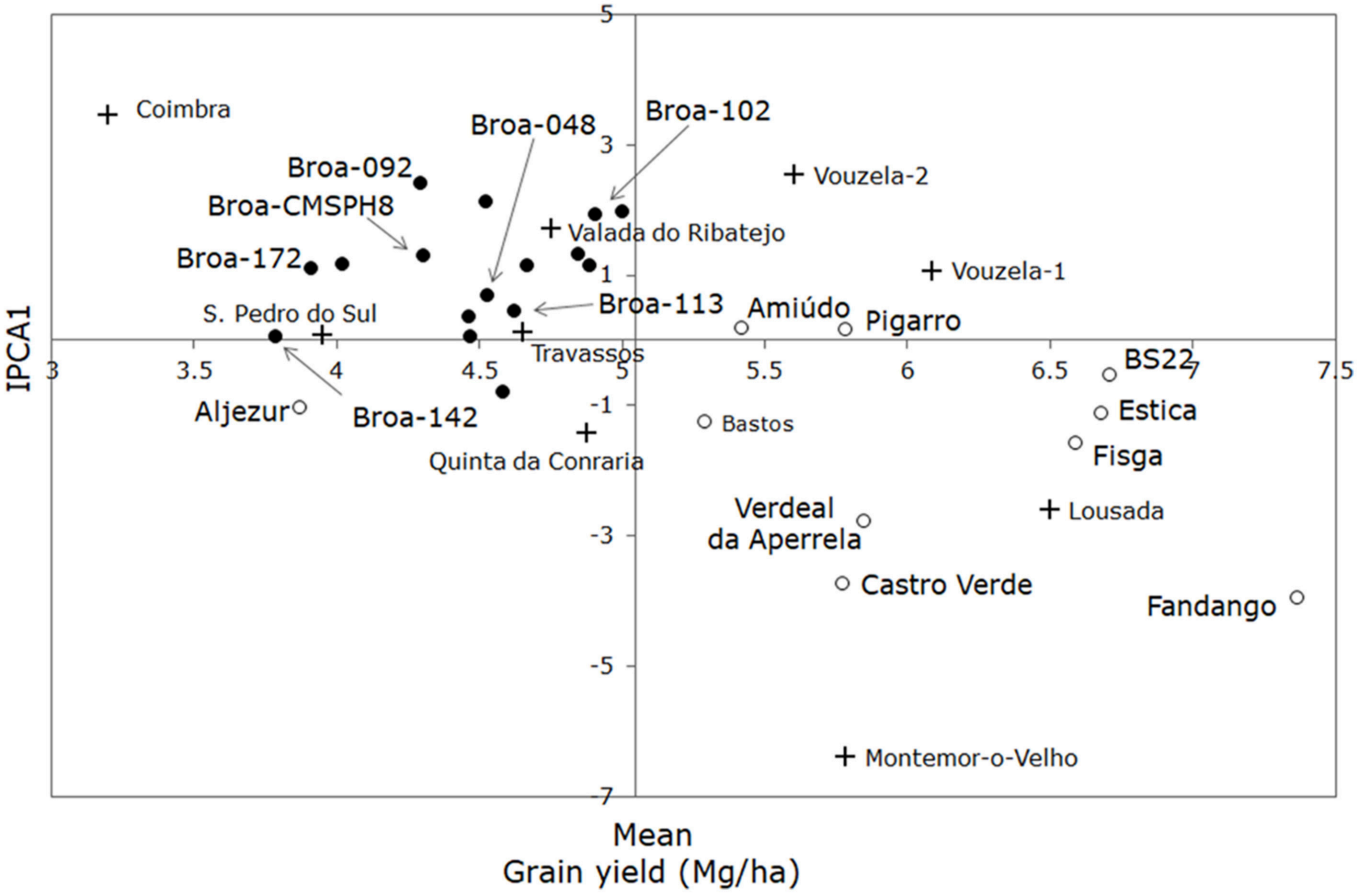
Biplot of mean grain yield against first principal component scores (IPCA1) of the Interaction Principal Component Analysis for 26 maize populations and nine tested environments. Legend: farmers' populations are depicted in black circles; participatory bred (PPB) populations and the outer group (*BS22(R)C6*) are depicted in white circles; tested environments are depicted in black crosses.

The AMMI ANOVA (Table 2) shows that population, environment, and the G × E interaction were significant (*P* < 0.05) for grain yield. From the total variation expressed as the sum of squares, the genotypes accounted for 28.12%, and the G × E interaction accounted for a 16.96% variation. The cross-validation identified AMMI1 as the optimal model; therefore, G × E was further partitioned into a single interaction principal component axis (IPCA) and model residual. The results of AMMI1 fitting for grain yield (Mg/ha) are illustrated on Figure 2. This biplot depicts both main effects for populations (G) and environments (E), on *x* axis, and G × E interaction, on *y* axis. Coordinates where the axes are crossing in the biplot correspond to the overall grain yield mean (5.05 Mg/ha) (on *x* axis) and no G × E interaction (on *y* axis). The vertical axis separates lower-yielding populations and the environments where the maize populations performed the worst on the left side from the higher-yielding populations and environments where populations performed the best on the right side. The population with the highest mean grain yield was *Fandango*, a participatory (PPB) bred maize population, and the population with the lowest mean grain yield was a farmers' maize population—*broa-142*. The horizontal axis separates all populations and environments into two groups with opposite interaction effects, and the strength of the interaction effects is depicted as the distance from the *x* axis to each environment; therefore, the Coimbra site has the strongest positive interaction effect on the populations' performance and the Montemor-o-Velho site the strongest negative interaction effect on the populations' performance. The positioning of a population close to a certain environment indicates the specific adaptation of those populations to those environments. Overall, all farmers' populations were low-yielding, with grain yield mean of 4.49 Mg/ha, value below the overall grain yield mean (5.05 Mg/ha), and with positive interaction effects with the Valada do Ribatejo, Travassos, and Coimbra sites; therefore, they are better adapted to those environments. Participatory bred populations with a long cycle until maturation (identified as late populations in Table S2), such as *Fandango, Estica, Fisga*, and *Verdeal da Aperrela*, had high grain yields (7.37 Mg/ha, 6.68 Mg/ha, 6.59 Mg/ha, and 5.85 Mg/ha, respectively) and performed better at environments such as the Montemor-o-Velho and Lousada sites.

**Table 2:** Additive Main effects and Multiplication Interaction (AMMI) analysis of variance for maize populations' grain yield tested in nine different environments.

**Source**	**Degrees of freedom**	**Mean square**	***P*-value**
Total	602	372.94	
Treatment	233	733.75	<0.001
Population	25	2525.58	<0.001
Environment	8	8719.55	<0.001
G × E ^a^	200	190.34	<0.05
IPCA1^b^	32^*^	486.70	<0.001^**^
Residual	168	133.89	0.723
Error	369	145.11	

a *G × E – Genotype-by-Environment interaction*.

b *IPCA1—first Interaction Principal Component Axis*.

* *Degrees of freedom assigned to IPCAs using Gollob's method (Gauch, 1992)*.

** *F ratio constructed using residual mean square as denominator*.

### Genetic diversity analysis

The molecular characterization of the populations was done using 20 microsatellites markers distributed evenly across the 10 maize chromosomes. The level of information retrieved from the markers used, calculated as the polymorphic information content (PIC), was, on average, 0.516. Overall the 20 microsatellites detected 114 different alleles, with an average of 5.7 alleles per marker (Table S4). Except for *broa-142*, from the farmers' populations, and Verdeal da Aperrela, from the participatory bred populations, both showing an excess of homozygous individuals (F_IS_ = 0.113 and F_IS_ = 0.093, respectively), no deviations from Hardy-Weinberg expectations were detected in the remaining 24 maize populations (Table S7).

The results of the genetic variability assessment within each population can be found in Table S7. When considering only the farmers' populations (*broa-x* populations), the lowest number of alleles and the lowest genetic diversity (H_E_) were found in population *broa-CMSPH8* (N_ar_ = 2.8; H_E_ = 0.405), whereas the highest values of both parameters were found in population *broa-113* (N_ar_ = 3.5; H_E_ = 0.549; Table S7). For comparison purposes, it is worth noting that the US population (*BS22(R)C6*) always showed values of the number of alleles and genetic diversity below the average values detected on the farmers' populations (Table S7). It was also revealed that the allelic richness (N_ar_) and genetic diversity (H_E_) were significantly lower on farmers' populations when compared to participatory bred populations (N_ar_ = 3.164 vs. N_ar_ = 3.692, H_E_ = 0.490 vs. H_E_ = 0.514) (Table 3).

**Table 3:** Differences in average values of N_ar_, H_O_, H_E_, and F_IS_ between farmers' populations and participatory bred (PPB) populations.

**Group**	**No. of populations**	**N_ar_**	**H_O_**	**H_E_**	**F_IS_**
Farmers' populations	16	3.164	0.487	0.490	0.008
PPB populations	9	3.692	0.514	0.544	0.055
*P*-value^*^		0.001	0.063	0.002	0.006

* *P-values obtained after 1,000 permutations*.

*N_ar_, allelic richness; H_O_, observed heterozygosity; H_E_, expected heterozygosity; F_IS_, inbreeding coefficient*.

Genetic differentiation between all pairs of populations was measured with pairwise F_ST_ estimates. All pairwise F_ST_ values were significantly different from zero at *P* < 0.05, except between *Estica* and *Fisga* populations.

The average genetic differentiation of farmers' populations was below the overall average (overall F_ST_ = 0.124 *vs*. farmers' populations F_ST_ = 0.099; Table S8).

The results from the (AMOVA; Excoffier et al., 1992) can be found in Table 4. AMOVA was used to partition the total microsatellite diversity: (1) among and within all populations; (2) among farmers' PPB populations, among populations within groups, and within all populations.

**Table 4:** Analysis of molecular variance (AMOVA) analysis for the partitioning of microsatellite diversity (1) among all populations and within populations, (2) among farmers' populations and participatory bred (PPB) populations, among populations within groups, and within all populations.

**Analysis**	**Source of variation**	**df^a^**	**Percentage of variation**	**ϕ-statistics^b^**	***P*-value (ϕ)^c^**
(1) All populations	Among populations	25	12.75	ϕ_ST_ = 0.127	<0.0001
	Within populations	1,534	87.25		
(2) Farmers' populations vs. PPB populations	Among groups	1	2.30	ϕ_CT_ = 0.023	<0.001
	Among populations within groups	23	10.29	ϕsc = 0.105	<0.0001
	Within populations	1,475	87.41	ϕ_ST_ = 0.126	<0.0001

a *df, Stands for degrees of freedom*.

b *ϕ-statistics: corresponds to an analogous to the Wright's F-statistics which measures the degree of genetic differentiation*.

c *P-value (ϕ): the level of significance of the ϕ-statistics was tested by non-parametric randomization tests using 10,000 permutations*.

The result from the AMOVA shows that most of the observed genetic variance (87.25%) can be explained by the heterogeneity that exists within each population—intra-population variability. Nevertheless, some degree of genetic differentiation exists between farmers' PPB populations with a ϕ_CT_ = 0.023 (*P*-value (ϕ) < 0.001; Table 4).

**Figure 3:**
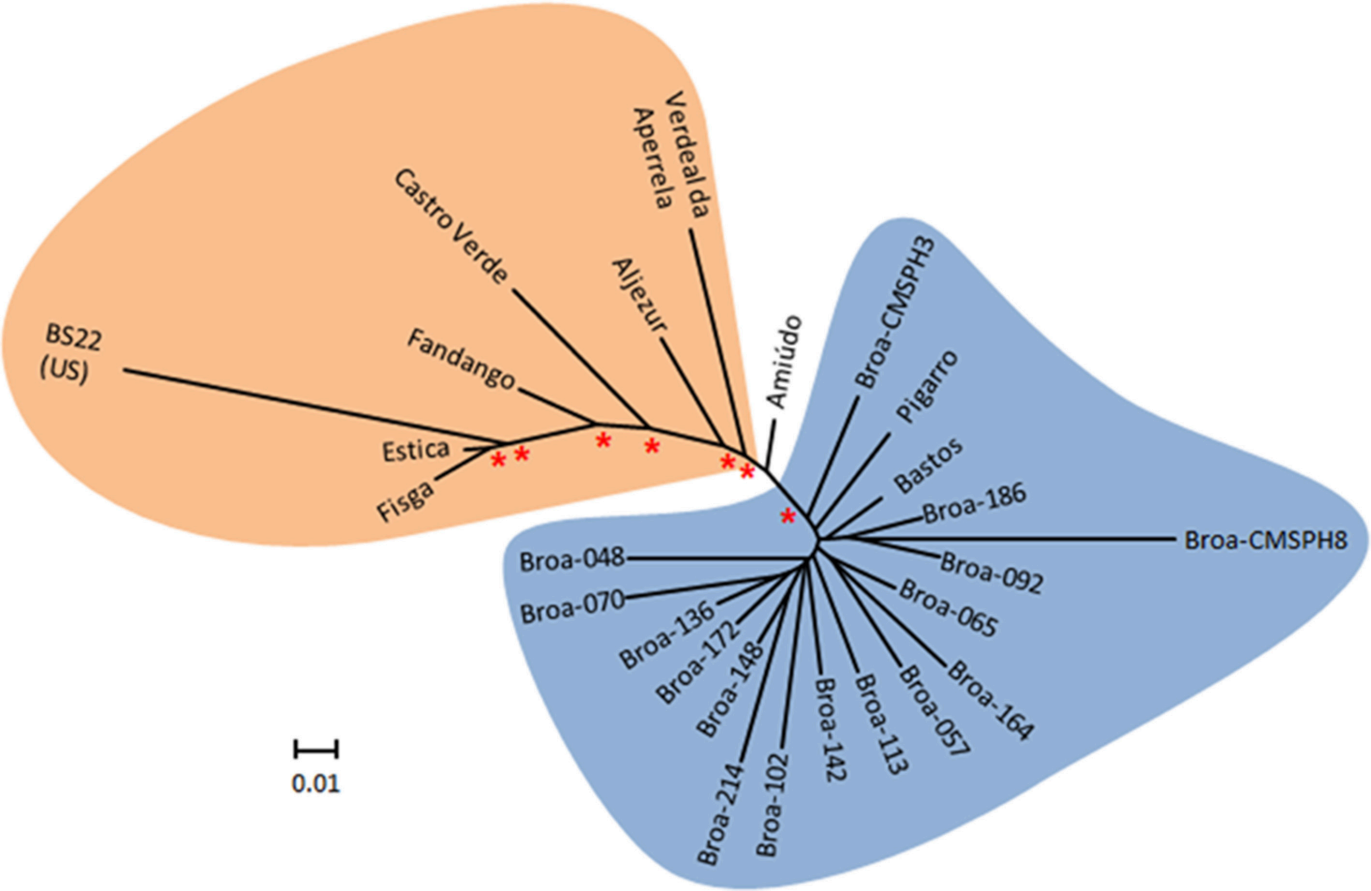
Fitch-Margoliash tree based on Cavalli-Sforza–Edwards' chord distances between 16 farmers' populations and 9 participatory bred (PPB) maize populations, plus the *BS22(R)C6* synthetic population from the US, abbreviated for *BS22* in the tree figure; bootstrap support values higher than 50% over 1,000 replicates are indicated with a red asterisk.

In the unrooted tree, all farmers' populations were placed on the same branch, clustered together with two participatory bred populations—*Pigarro* and *Bastos*. Moreover, the farmers' populations were placed further away from the populations with a US genetic background—*BS22(R)C6, Fandango, Estica*, and *Fisga* (Figure 3).

The average genetic distance between all populations was 0.104, with the minimum distance observed between two participatory bred populations (*Estica* and *Fisga, D*_*CSE*_ = 0.021) and the maximum distance observed between a farmers' population—*broa-CMSPH8*—and the outer group population—*BS22(R)C6*—(*D*_*CSE*_ = 0.281; Figure 3, Table S9).

The existence of a genetic structure within the overall set of maize populations was investigated using a model-based clustering method implemented in STRUCTURE software (Pritchard et al., 2000). The highest ΔK value was observed for K = 2 (for K = 2, ΔK = 336.156, a value considerably bigger than the subsequent ΔK value for K = 3, ΔK = 67.031) and therefore two gene pools were considered to be the optimal solution. The proportion of membership of each gene pool in the 30 individual plants analyzed per population was retrieved from the run with the highest average estimates of the likelihood of the data, conditional on a given number of clusters, *ln*[Pr(X|K)].

From the 16 farmers' populations analyzed, all were predominantly build of gene pool A (Figure S1, gene pool A in blue), averaging a proportion of membership of 93.3 ± 9.6%.

## Discussion

Given the previous successful Portuguese experience in participatory maize breeding and to promote the use of the maize populations collected from a *broa*-producing region, this work aimed to develop decision-making tools to support the establishment of a new participatory maize quality-oriented breeding program in the country.

### Maize populations' quality characterization

The detailed characterization performed in the present study allowed for the identification of two main quality-based groups, and an outlying population, *Amiúdo*. *Amiúdo* clearly differed from the remaining maize populations in terms of its higher carotenoids level and lower levels in *p*-coumaric and ferulic acids. The different quality-based groups detected by cluster analysis were in agreement with the results obtained from PCA: 14 out of the 16 farmers' populations analyzed were placed in the same quality-group, named quality-group I, which corresponds to 87.5% of the farmers' populations (*broa-x* populations), with the exception of *broa-092* and *broa-102* populations; *broa-x* populations were essentially separated from the non-*broa-x* populations by their higher protein and fiber content, their lower levels of total carotenoids, α- and δ-tocopherol, and volatile aldehydes, as well as by their lower breakdown viscosities values. Populations belonging to quality-group I had on average 12.18% protein, a value slightly above the average reported for maize kernel (8–11% of protein, % w/w, Fao, 1992) but similar to the values (12.73–13.33%) previously reported by Vaz Patto et al. (2009) using an extended number of Portuguese maize populations. Quality group I populations also presented on average 2.36% in fiber, which is similar to the value reported for maize kernel (2% fiber, % w/w, Fao, 1992; 2.59–2.61% in Vaz Patto et al., 2009). The populations from quality-group I had lower breakdown viscosities when compared with the populations from the other quality-group, which were composed mainly of non-*broa-x* populations. Breakdown viscosity (BD) is calculated as the difference between the peak (maximum) and the trough (minimum) viscosities obtained during the RVA heating-cooling cycle. Breakdown viscosity is a measure of how easily the swollen starch granules can be disrupted after peak viscosity is reached during the Rapid Visco Analyser (RVA) heating-cooling cycle (Wani et al., 2012). Since the breakdown viscosity is the result of the disintegration of starch granules, this value suggests the degree of starch stability during cooking (Wani et al., 2012). Julianti et al. (2015), when studying different composite flour formulations, observed that by increasing the proportion of soybean flour, a flour rich in protein, the breakdown viscosity measured during the RVA heating-cooling cycle decreased. In the present work protein content and breakdown viscosity values are shown to have a strongly negative correlation between them. Related to what was discussed by Julianti et al. (2015), one of the possible explanations for the lower breakdown viscosities values observed in this current work in farmers' populations (*broa-x* populations) is the higher level of protein usually detected on those materials compared to the values obtained for the majority of non-*broa-x* populations.

It is known that the chemical composition of flour will influence the food texture and aroma (Collar et al., 2015; Shobha et al., 2015). Additionally, the maize populations that produce better-quality *broa* have higher protein values and lower breakdown values when compared to commercial maize varieties (Brites et al., 2010). The higher protein contents can probably induce increased amounts of flour water absorption ratio and corresponding higher bread moisture. In fact, the crumb moisture was been identified (Carbas et al., 2016) as a relevant attribute for consumer acceptability of *broa*.

Taking all that into consideration, according to the values of protein and breakdown viscosity obtained for traditional maize populations in the current work, and previously by Vaz Patto et al. (2009), one can argue that for maize populations used for *broa* production the optimal range values will be 12–13% of protein, and breakdown viscosity values of 82-300 cPoise.

Besides the basic nutritional value and pasting behavior-related traits also previously studied in Vaz Patto et al. (2009), in the current work, quality traits that might influence consumers' preferences/choices, such as volatile compounds related to aroma and health-related compounds such as carotenoids, tocopherols, and phenolic compounds, were also analyzed.

Vitamin A, as provitamin A carotenoids, and vitamin E, as tocopherols, are the predominant fat-soluble vitamins found in maize kernels (Nuss and Tanumihardjo, 2010). Moreover, the health benefits of grain products have also been associated with the antioxidant properties of the phenolic compounds found in grains (Bonoli et al., 2004). Carotenoids are a diverse family of yellow-orange pigments (Nuss and Tanumihardjo, 2010), and even though previous reports showed that grain color is not necessarily correlated with a provitamin A concentration of yellow and orange maize (e.g., Harjes et al., 2008), in the current work a strong positive correlation between the total carotenoid content and flour yellowness was detected.

Within the antioxidant phenolic compounds, ferulic acid is predominant in maize kernel, mainly present in the bound form (Adom and Liu, 2002), with *p*-coumaric acid also widely found in maize (Pei et al., 2016). Within the present study quality-group I, composed mainly by *broa-x* populations, a substantial range of variation could be found for flour yellowness and total carotenoids, and for the two individual phenolic compounds analyzed—*p*-coumaric acid and ferulic acid. This indicates that further improvement to increase the attractiveness of food formulations based on the populations within that quality-group, and specifically for those traits, where variation can still be found, is still possible. Indeed, some of these antioxidant compounds may reduce the retrogradation and improve starch qualities (Beta and Corke, 2004; Zhu et al., 2009; Siriamornpun et al., 2016), or influence the formation of dough texture (Klepacka and Fornal, 2006), a very important parameter in defining bread quality (Matos and Rosell, 2012).

Maize kernel nutritional composition can varies due various factors such as the genotype, environmental conditions, and processing (Prasanthi et al., 2017). In the future, the study of G × E interaction for quality traits should also be undertaken since genotype-by-environment interaction are known to affect some quality traits (e.g., Malvar et al., 2008; Revilla et al., 2015). This study would allow us not only to test the significance of the G × E on the presently considered quality traits, but also to compare, for each trait, the proportion of explained variance by the G × E term with respect to the genotype main effects.

Because data acquisition for the quality traits accessed in this study is very expensive and time consuming in the present work genotype-by-environment analysis was only performed at an agronomic level. Nevertheless, even with quality data from only one common-garden experiment, the results obtained from the multivariate analysis allowed us to highlight the similarities that exist among farmers' populations, as well as to identify the quality traits that discriminate them.

### Maize populations' agronomic performance

Multi-location field trials were established across different farming systems in order to accurately estimate the agronomic performance and evaluate the agronomic potential of the farmers' maize populations. An Additive Main effects and Multiplicative Interaction (AMMI) method was implemented to identify maize populations with broader stability (i.e., lower variation across locations) or specific adaptability to the tested locations, and to evaluate potential new locations for the quality-oriented breeding program in the country. According to Furtado Ferreira et al. (2006), an undesirable population will have low stability associated with low productivity; therefore, the ideal population is one with high productivity and IPCA1 values close to zero (stable across environments).

The lower the IPCA1 value (in absolute values), the lower its contribution to the G × E interaction; therefore, the more stable the agronomic behavior of the population. On average, and in terms of grain production, the farmers' populations analyzed in the present work had a broader stability value when compared to all the maize populations (|IPCA1|_FARMERS_ = 1.124 vs. |IPCA1|_OVERALL_ = 1.635). However, the results also showed that all farmers' populations were low-yielding (4.49 Mg/ha, on average), performing better in environments such as the Valada do Ribatejo (organic production), Travassos, or Coimbra sites.

In conclusion, the agronomic evaluation allowed for the identification of the most appropriate locations where selection activities should be pursued if increasing grain yield and/or ear weight is among the breeding objectives in a quality-oriented participatory maize breeding program. Moreover, that choice can be fine-tuned according to the maize populations under selection. Of course, other factors, such as local support/interest from both farmers and local institutions (e.g., municipality and farmers' associations) must be taken into consideration when choosing the location for this kind of participatory research (Vaz Patto et al., 2013). In addition, the end product to be produced (maintaining the ethnic maize-based bread entity or extending it to other novelty food products) may influence the choice of the location as well as the particular populations that are more suitable due to their quality traits. In this way, if a population or a group of populations selected for a quality objective/end-use behaves better in a particular environment, this might be the best environmental choice. An extra factor to keep in mind for these decisions: should we consider the quality certification of the end product? For example, if we were to consider the Portuguese ethnic maize-based bread as a value-added product by adding a certification, according to the European Union (EU) agricultural product quality policy [such as protected designations of origin (PDOs), protected geographical indications (PGIs), or traditional speciality guaranteed (TSG) (https://ec.europa.eu/agriculture/quality_en; accessed August 30th 2017)], this possibility of certification might have profound implications on the organization of the breeding program. Not only geographic implications [selection of the site(s) for PPB implementation], if one wants to select for a particular environment, but also on the breeding design/crosses allowed (intra-population selection, selection of one population vs. inter-populations crosses, selection of several populations).

### Phenotypic and molecular characterization data integration

One of the proposed objectives of this study was to build decision-making tools for an accurate population selection within a quality-oriented participatory breeding program. This was achieved by complementing a precise agronomic and quality description with a more thorough molecular characterization.

For example, in the case in which we need to start from either one particular population (intra-population selection) or from several populations (inter-populations crosses), molecular information such as that gathered in this study acts as an effective extra decision-making tool to evaluate and compare the genetic resources available to breeders. As already pointed out by Reif et al. (2003), simple sequence repeat markers provide a valuable tool for grouping germplasm and are a good complement to field trials for identifying groups of genetically similar germplasm.

The genetic diversity/distance calculated between potential crossing parents can be chosen to assure the highest possible diversity within a cross (Tuvesson et al., 2007), to plan useful gene combinations, increasing the performance through increased heterosis (Reif et al., 2003), or to add new variation to the breeding program in a controlled fashion (Tuvesson et al., 2007).

In the present work, based on the genetic distances and genetic structure of the maize populations, two main clusters could be identified that in a systematic manner separated the maize populations with a known US genetic background from the other maize populations. One of the clusters contained all the *broa-x* populations together with two participatory bred populations derived from two traditional maize populations (*Pigarro* and *Bastos*). The quality-group I, which is composed mainly of farmers' populations (14 *broa-x* populations), plus one participatory bred population (*Bastos*), is almost identical to this genetic-based cluster (only *Pigarro* is not included). We also observed that the maize flour from the majority of the *broa-x* Portuguese populations, evaluated at the Coimbra site, had higher levels of protein and fiber and lower levels of α- and δ-tocopherols, associated with a lower breakdown viscosity values when compared to the maize populations of quality-group II.

For illustration purposes, in the case of a quality oriented breeding program for maize bread using the Portuguese populations, one of the breeding objectives to be pursued could focus on increasing the agronomic performance of the populations and tocopherol levels (α- and δ-tocopherol content) that are limiting on this germplasm, but without compromising the protein content or increasing viscosity. An increase in maize vitamin E levels, as tocopherols, can elevate its nutritional value by enhancing their role as antioxidants (Nuss and Tanumihardjo, 2010). As an example, one can improve the α-tocopherol levels on these Portuguese populations by using as a donor parent the maize population with the highest α-tocopherol levels (*Fandango*; 123.64 μg/g fat basis; a population with a known US genetic background). The cross with the *Fandango* population, genetically distant from the *broa-x* populations, may promote heterosis and consequently a higher agronomic performance of the resulting hybrid.

As in the described example, the knowledge generated from both phenotypic and genotypic analysis will aid in deciding future breeding activities and genetic resources management. As for bread making and other end uses, the same decision-making process could be used to select the initial populations, breeding approaches, and optimal breeding locations. At present, existing information is already in use to identify potential maize open-pollinated populations as parental lines to generate better-performing population hybrids with increased content in tocopherols and total free phenolic compounds, decreased content in volatile aldehydes, and decreased overall viscosity. This information was compiled separately according to the populations' kernel color (white kernel vs. non-white kernel) since kernel color has been linked to consumer acceptance (Ranum et al., 2014) and also appears to be important for Portuguese maize bread consumer choices (Carbas et al., 2016).

Through the integration of the different levels of information available, more informed choices are optimizing the use of resources and improving the efficiency of participatory breeding activities.

## Author contributions

MLA performed the DNA isolation and the SSR genotyping, the analysis of the molecular and quality data, and drafted the manuscript. CB and MP participated in the establishment of the field trials, and performed the agronomic characterization. BC and MB performed the quality characterization. PMRM-M coordinated the field trials and performed the agronomic characterization. CB and MRB coordinated and participated in the acquisition of quality data, and participated in the manuscript revision. JG performed the analysis of the agronomic data, and critically participated in the manuscript revision. ZŠ participated in the analysis of the molecular and phenotypic data, and critically participated in the manuscript revision. MCVP designed and coordinated the study and participated in the drafting and revising of the manuscript. All authors read and approved the final manuscript.

### Conflict of interest statement

The authors declare that the research was conducted in the absence of any commercial or financial relationships that could be construed as a potential conflict of interest. The reviewer PR and handling Editor declared their shared affiliation.
