# Supplementary material for: Setting Up Decision-Making Tools toward a Quality-Oriented Participatory Maize Breeding Program
Source: Front Plant Sci. 2017 Dec 22;8:2203. doi: 10.3389/fpls.2017.02203 (PMC5744637; doi:10.3389/fpls.2017.02203)
Supplement: Supplementary file 1 [file Table1.docx]

***Supplementary Material***

**Setting up decision-making tools towards a quality-oriented participatory maize breeding program**

**Authors**

Mara Lisa Alves^1^, Cláudia Brites^2^, Manuel Paulo^2^, Bruna Carbas^3^, Maria Belo^1^, Pedro Mendes-Moreira^2^, Carla Brites^3^, Maria do Rosário Bronze^1, 4, 5^, Jerko Gunjača^6,7^, Zlatko Šatović^6,7^, Maria Carlota Vaz Patto^1^*

**Correspondence**

*Corresponding author: [cpatto@itqb.unl.pt](mailto:cpatto@itqb.unl.pt)

**Table S1.** Maize populations under study, seed source, information on previous selection efforts, brief morphological characterization of the populations (endosperm type, grain color and population maturation), populations’ geographic origin and geographic coordinates of the populations from the collection mission.

| Source | Population | Population Type | Farmers’ selection | Selection objective | Endosperm type | Grain color | Maturation | Geographic origin | Geographic Coordinates (Latitude / Longitude) | Altitude (masl^3^) |
| --- | --- | --- | --- | --- | --- | --- | --- | --- | --- | --- |
| Farmer | Broa-048 | Traditional population | Yes | Wide and longer ears | flint | yellow | early | Central Portugal | 40º50'32''N / 7º56'14''W | 471 |
| Farmer | Broa-057 | Traditional population | Yes | Best and bigger ears | flint | white | early | Central Portugal | 40º54'49''N / 7º58'22''W | 502 |
| Farmer | Broa-065 | Traditional population | NA | NA | flint | white | early | Central Portugal | 40º57'05''N / 7º54'52''W | 876 |
| Farmer | Broa-070 | Traditional population | Yes | Best ears full of grains | flint | white | early | Central Portugal | 40º41'48''N / 8º04' 58''W | 567 |
| Farmer | Broa-092 | Traditional population | Yes | Best ears | flint | white | early | Central Portugal | 40º38'02''N / 8º03'42''W | 429 |
| Farmer | Broa-102 | Traditional population | Yes | Earliness, smaller plants, smaller grain and full ears | flint | yellow | early | Central Portugal | 40º39'55''N / 7º59'00''W | 424 |
| Farmer | Broa-113 | Traditional population | Not currently | Bigger and fascinated ears | flint | orange | early | Central Portugal | 40º45'04''N / 7º34'26''W | 609 |
| Farmer | Broa-136 | Traditional population | Not currently | Bigger ears | flint | white | early | Central Portugal | 40º19'30''N / 7º41'10''W | 795 |
| Farmer | Broa-142 | Traditional population | Not currently | Fasciated ears | flint | white | early | Central Portugal | 40º28'33''N / 7º29'03''W | 801 |
| Farmer | Broa-148 | Traditional population | NA^2^ | NA | flint | yellow | early | Central Portugal | 40º31'31''N / 7º34'13''W | 459 |
| Farmer | Broa-164 | Traditional population | Yes | Wider ears | flint | orange | early | Central Portugal | 40º39'01''N / 7º24'32''W | 423 |
| Farmer | Broa-172 | Traditional population | NA | NA | flint | white | early | Central Portugal | 40º19'55''N / 7º50'33''W | 269 |
| Farmer | Broa-186 | Traditional population | Yes | Smaller plants and bigger ears | flint | white | early | Central Portugal | 40º25'19''N / 7º55'19''W | 338 |
| Farmer | Broa-214 | Traditional population | Yes | Earliness, bigger ears with no diseases or lodging | flint | white | early | Central Portugal | 40º49'43''N / 8º13'06''W | 660 |
| Farmer | Broa-CMSPH3 | Traditional population | NA | NA | flint | white | early | Central Portugal | 40º16'13.74''N / 8º16'49.97''W | 130 |
| Farmer | Broa-CMSPH8 | Traditional population | NA | NA | flint | white | early | Central Portugal | 41º07'1.75''N / 8º05'12.75''W | 321 |
| PPB^1^ | Amiúdo | Traditional population |  | Yield | flint | yellow | early | Northern Portugal |  |  |
| PPB | Bastos | Traditional population |  | NA | flint | white | early | Northern Portugal |  |  |
| PPB | Pigarro | Traditional population |  | Bigger years | flint | white | early | Northern Portugal |  |  |
| PPB | Verdeal da Aperrela | Traditional population |  | NA | flint | white | late | Northern Portugal |  |  |
| PPB | Aljezur | Traditional population |  | NA | flint | yellow | early | Southern Portugal |  |  |
| PPB | Castro Verde | Traditional population |  | Bigger years | flint | yellow | late | Southern Portugal |  |  |
| PPB | Estica | Synthetic population |  | Longer years | dent | yellow | Late | 80% USA, 20% Portugal |  |  |
| PPB | Fisga | Synthetic population |  | Prolificacy | dent | yellow | Late | 80% USA, 20% Portugal |  |  |
| PPB | Fandango | Synthetic population |  | Yield | dent | yellow | Late | 80% USA, 20% Portugal |  |  |
| USA | BS22(R)C6 | Synthetic population |  | yield, root and stalk strength | dent | yellow | early | USA |  |  |

*^1^ PPB stands for Portuguese participatory maize breeding program*

*^2^ NA stands for information not available*

*^3^ masl stands for meters above sea level*
