# Supplementary material for: Setting Up Decision-Making Tools toward a Quality-Oriented Participatory Maize Breeding Program
Source: Front Plant Sci. 2017 Dec 22;8:2203. doi: 10.3389/fpls.2017.02203 (PMC5744637; doi:10.3389/fpls.2017.02203)
Supplement: Supplementary file 2 [file Table2.docx]

***Supplementary Material***

**Setting up decision-making tools towards a quality-oriented participatory maize breeding program**

**Authors**

Mara Lisa Alves^1^, Cláudia Brites^2^, Manuel Paulo^2^, Bruna Carbas^3^, Maria Belo^1^, Pedro Mendes-Moreira^2^, Carla Brites^3^, Maria do Rosário Bronze^1, 4, 5^, Jerko Gunjača^6,7^, Zlatko Šatović^6,7^, Maria Carlota Vaz Patto^1^*

**Correspondence**

*Corresponding author: cpatto@itqb.unl.pt

**Table S2.** Location, soil and climate characterization of the field trials sites.

|  | |  |  | Soil conditions^1^ | | | | Climatic conditions^2^ – rainfall (mm) and average temperature (ºC) | | | | | | |
| --- | --- | --- | --- | --- | --- | --- | --- | --- | --- | --- | --- | --- | --- | --- |
| Location | Year^3^ | Geographic coordinates  Latitude / Longitude | Altitude (masl^4^) | Dominant soil group | Topsoil pH | Topsoil organic carbon (% weight) | Available water storage capacity (mm) | Accumulated rainfall (mm) | Temp May (ºC) | Temp June (ºC) | Temp July (ºC) | Temp August (ºC) | Temp September (ºC) | Temp October (ºC) |
| Lousada | 2010 | 41º14'7.8''N / 8º18'11.1''W | 198 | Humic cambisols | 5.3 | 2.72 | 50 | 322.0 | 16 | 19 | 24 | 23 | 21 | 15 |
| Travassos | 2010 | 41°25'21.53"N / 7°50'42.91"W | 636 | Humic cambisols | 5.3 | 2.72 | 50 | 321.0 | 14 | 19 | 24 | 23 | 20 | 14 |
| S. Pedro do Sul | 2010 | 40º47'7.91''N / 8º1'35.17''W | 475 | Humic cambisols | 5.3 | 2.72 | 50 | 325.5 | 14 | 19 | 24 | 23 | 20 | 14 |
| Vouzela-1 | 2010 | 40°42'35.1"N / 8°08'01.6"W | 461 | Humic cambisols | 5.3 | 2.72 | 50 | 323.5 | 16 | 19 | 24 | 23 | 20 | 15 |
| Vouzela-2 | 2010 | 40º42'25.31''N / 8º08'30.25''W | 450 | Humic cambisols | 5.3 | 2.72 | 50 | 323.5 | 16 | 19 | 24 | 23 | 20 | 15 |
| Quinta da Conraria | 2010 | 40º10'35.66''N / 8º23'44.80''W | 40 | Dystric regosols | 5.1 | 1.39 | 150 | 284.5 | 16 | 21 | 24 | 25 | 22 | 17 |
| Montemor-o-Velho | 2010 | 40º10'4.82''N / 8º41'14.84''W | 3 | Eutric fluvisols | 7.2 | 0.86 | 150 | 199.5 | 16 | 19 | 22 | 23 | 21 | 17 |
| Valada do Ribatejo | 2010 | 39º6'2.30''N / 8º47'30.27''W | 5 | Eutric fluvisols | 7.2 | 0.86 | 150 | 184.5 | 18 | 21 | 24 | 25 | 23 | 17 |
| Coimbra | 2010 | 40°13'0.22"N / 8°26'47.69"W | 21 | Chromic cambisols | 7.1 | 0.65 | 15 | 284.5 | 16 | 21 | 24 | 25 | 22 | 17 |
| Coimbra | 2009 | 40°13'0.22"N / 8°26'47.69"W | 21 | Chromic cambisols | 7.1 | 0.65 | 15 | 192.0 | 18 | 21 | 21 | 22 | 22 | 19 |

*^1^ Data from FAO/IIASA/ISRIC/ISSCAS/JRC, 2012. Harmonized World Soil Database (version 1.2), available trough <http://www.fao.org/soils-portal/soil-survey/soil-maps-and-databases/harmonized-world-soil-database-v12/en/>*

*^2^ Data from (IPMA, I. P.).* – *Instituto Português do Mar e da Atmosfera, I. P <https://www.ipma.pt/en/oclima/monitorizacao>; Accumulated rainfall and average temperatures registered during maize growing season (period extended from May until October).*

*^3^ 2010 field trials were used for agronomic evaluation; 2009 field trial was used for quality evaluation*

*^4^ masl stands for meters above sea level*

**References**

FAO/IIASA/ISRIC/ISSCAS/JRC, 2012. Harmonized World Soil Database (version 1.2). FAO, Rome, Italy and IIASA, Laxenburg, Austria.

Instituto Português do Mar e da Atmosfera, I. P. (IPMA, I. P.). Climate Monitoring. Accessed February 2, 2017. <https://www.ipma.pt/en/oclima/monitorizacao/>
