# Supplementary material for: Setting Up Decision-Making Tools toward a Quality-Oriented Participatory Maize Breeding Program
Source: Front Plant Sci. 2017 Dec 22;8:2203. doi: 10.3389/fpls.2017.02203 (PMC5744637; doi:10.3389/fpls.2017.02203)
Supplement: Supplementary file 3 [file Table3.docx]

***Supplementary Material***

**Setting up decision-making tools towards a quality-oriented participatory maize breeding program**

**Authors**

Mara Lisa Alves^1^, Cláudia Brites^2^, Manuel Paulo^2^, Bruna Carbas^3^, Maria Belo^1^, Pedro Mendes-Moreira^2^, Carla Brites^3^, Maria do Rosário Bronze^1, 4, 5^, Jerko Gunjača^6,7^, Zlatko Šatović^6,7^, Maria Carlota Vaz Patto^1^*

**Correspondence**

*Corresponding author: [cpatto@itqb.unl.pt](mailto:cpatto@itqb.unl.pt)

**Table S3.** List of agronomic traits evaluated per plot basis, abbreviation and respective description.

| Trait | Abbreviation | Units/Scale | Description |
| --- | --- | --- | --- |
| Ear weight | EW | gram (g) | Ear weight, adjusted to 15% of grain moisture. Measure by averaging the weight of 4 shelled ears per plot. |
| Grain yield^1^ | Y | kilogram/hectare (kg/ha) | Grain yield adjusted to 15% moisture. Formula: Grain yield = Ear weight × (Grain weight/Ear weight) × (100%–% moisture at harvest)/ (100%–15% moisture). Grain weight and ear weight taken from 4 shelled ears. |

*^1^Grain yield adjusted to 15% of moisture was calculated according to Moreira et al. (2008)*
