# Supplementary material for: Setting Up Decision-Making Tools toward a Quality-Oriented Participatory Maize Breeding Program
Source: Front Plant Sci. 2017 Dec 22;8:2203. doi: 10.3389/fpls.2017.02203 (PMC5744637; doi:10.3389/fpls.2017.02203)
Supplement: Supplementary file 4 [file Table4.docx]

***Supplementary Material***

**Setting up decision-making tools towards a quality-oriented participatory maize breeding program**

**Authors**

Mara Lisa Alves^1^, Cláudia Brites^2^, Manuel Paulo^2^, Bruna Carbas^3^, Maria Belo^1^, Pedro Mendes-Moreira^2^, Carla Brites^3^, Maria do Rosário Bronze^1, 4, 5^, Jerko Gunjača^6,7^, Zlatko Šatović^6,7^, Maria Carlota Vaz Patto^1^*

**Correspondence**

*Corresponding author: cpatto@itqb.unl.pt

**Table S4.** List of 20 microsatellite loci, their repeat motifs, chromosomal bin positions, and allelic diversity within 26 maize populations (N = 780).

| No | Marker | Repeat motif | Bin location | Range (bp^1^) | N_a_^2^ | PIC^3^ |
| --- | --- | --- | --- | --- | --- | --- |
| 1 | nc007 | (CCT) | 5.01 | 143 – 158 | 6 | 0.701 |
| 2 | phi059 | (ACC) | 10.02 | 142 – 160 | 6 | 0.416 |
| 3 | phi065 | CACTT | 9.03 | 131 – 156 | 5 | 0.501 |
| 4 | phi084 | (GAA) | 10.03-10.04 | 148 – 160 | 4 | 0.442 |
| 5 | umc1065 | (ACA)17 | 2.06 | 128 – 164 | 13 | 0.832 |
| 6 | umc1134 | (AGC) | 7.03 | 79 – 91 | 7 | 0.491 |
| 7 | umc1139 | (GAC)4 | 8.01 | 146 – 158 | 3 | 0.283 |
| 8 | umc1267 | (CGG)4 | 9.03-9.04 | 113 – 122 | 4 | 0.582 |
| 9 | umc1329 | (GCC)7 | 4.06 | 77 – 86 | 3 | 0.426 |
| 10 | umc1425 | (TCA)4 | 3.04 | 119 – 131 | 5 | 0.368 |
| 11 | umc1431 | (GCA)5 | 1.09 | 132 – 138 | 3 | 0.54 |
| 12 | umc1689 | (GCG)5 | 1.05 | 137 – 146 | 4 | 0.403 |
| 13 | umc1690 | (GCA)4 | 3.07 | 82 – 94 | 4 | 0.425 |
| 14 | umc1777 | (CTG)4 | 8.05 | 113 – 125 | 5 | 0.412 |
| 15 | umc1787 | (CGG)4 | 7.02 | 80 – 89 | 3 | 0.411 |
| 16 | umc2030 | (CGA)4 | 2.04 | 112 – 124 | 5 | 0.574 |
| 17 | umc2059 | (CAG)8 | 6.08 | 122 – 146 | 8 | 0.727 |
| 18 | umc2196 | (CCG) | 6.01 | 115 – 133 | 6 | 0.552 |
| 19 | umc2216 | (TC)10 | 5.06 | 118 – 134 | 9 | 0.492 |
| 20 | umc2281 | (GTCC)5 | 4.03 | 152 – 204 | 11 | 0.734 |
|  | Average |  |  |  | 5.7 | 0.516 |
|  | Total |  |  |  | 114 |  |

*^1^ bp* – *base pairs*

*^2^ N_a_* – *total number of alleles*

*^3^ PIC* – *Polymorphism Information Content*
