# Supplementary material for: Setting Up Decision-Making Tools toward a Quality-Oriented Participatory Maize Breeding Program
Source: Front Plant Sci. 2017 Dec 22;8:2203. doi: 10.3389/fpls.2017.02203 (PMC5744637; doi:10.3389/fpls.2017.02203)
Supplement: Supplementary file 5 [file Table5.docx]

***Supplementary Material***

**Setting up decision-making tools towards a quality-oriented participatory maize breeding program**

**Authors**

Mara Lisa Alves^1^, Cláudia Brites^2^, Manuel Paulo^2^, Bruna Carbas^3^, Maria Belo^1^, Pedro Mendes-Moreira^2^, Carla Brites^3^, Maria do Rosário Bronze^1, 4, 5^, Jerko Gunjača^6,7^, Zlatko Šatović^6,7^, Maria Carlota Vaz Patto^1^*

**Correspondence**

*Corresponding author: [cpatto@itqb.unl.pt](mailto:cpatto@itqb.unl.pt)

**Table S5.** Pearson correlation coefficients among 17 quality traits calculated from a common-garden experiment (Coimbra, 2009) for 26 maize populations analyzed.

|  | PR | FI | FT | BD | SB1 | FV | PV | TV | *b** | TCC | AT | DT | GT | PH | CU | FE | AL |
| --- | --- | --- | --- | --- | --- | --- | --- | --- | --- | --- | --- | --- | --- | --- | --- | --- | --- |
| PR | 1.000 | *** | ns | *** | ns | ns | ** | ns | ns | ns | *** | *** | ns | ns | ns | ns | ** |
| FI | **0.954** | 1.000 | ns | *** | ns | ns | * | ns | ns | ns | *** | *** | ns | ns | ns | ns | ** |
| FT | -0.138 | 0.132 | 1.000 | ns | ns | ns | ns | ns | * | * | ns | ns | ns | ns | ns | ns | ns |
| BD | **-0.752** | **-0.711** | 0.059 | 1.000 | * | ns | *** | ns | ns | ns | ** | * | ns | ns | ns | ns | ns |
| SB1 | -0.172 | -0.147 | 0.079 | **0.410** | 1.000 | *** | *** | *** | ns | ns | ns | ns | ns | ns | * | * | ns |
| FV | -0.085 | -0.057 | 0.101 | 0.377 | **0.990** | 1.000 | *** | *** | ns | ns | ns | ns | ns | ns | * | * | ns |
| PV | **-0.505** | **-0.460** | 0.115 | **0.890** | **0.723** | **0.727** | 1.000 | *** | ns | ns | ns | ns | ns | ns | ns | ns | ns |
| TV | 0.161 | 0.191 | 0.149 | 0.250 | **0.863** | **0.925** | **0.664** | 1.000 | ns | ns | ns | ns | * | ns | * | ns | * |
| *b** | -0.161 | -0.279 | **-0.417** | 0.130 | 0.330 | 0.295 | 0.182 | 0.172 | 1.000 | *** | ns | ns | ns | * | ns | ns | ns |
| TCC | -0.261 | -0.372 | **-0.427** | 0.188 | 0.279 | 0.230 | 0.180 | 0.073 | **0.985** | 1.000 | ns | * | ns | * | ns | ns | ns |
| AT | **-0.764** | **-0.786** | -0.133 | **0.569** | -0.045 | -0.124 | 0.285 | -0.329 | 0.248 | 0.371 | 1.000 | *** | * | ns | ns | ns | * |
| DT | **-0.693** | **-0.719** | -0.157 | **0.485** | -0.028 | -0.118 | 0.210 | -0.349 | 0.290 | **0.423** | **0.869** | 1.000 | * | ns | ns | ns | ** |
| GT | -0.230 | -0.247 | -0.126 | 0.069 | -0.257 | -0.305 | -0.137 | **-0.404** | 0.257 | 0.324 | **0.443** | **0.483** | 1.000 | ns | ns | ns | * |
| PH | 0.084 | 0.186 | 0.331 | -0.025 | 0.021 | 0.064 | 0.063 | 0.174 | **-0.434** | **-0.448** | -0.125 | -0.143 | 0.169 | 1.000 | ns | ns | ns |
| CU | -0.309 | -0.226 | 0.309 | 0.087 | **-0.395** | **-0.418** | -0.138 | **-0.437** | -0.228 | -0.206 | 0.116 | 0.023 | -0.022 | -0.037 | 1.000 | ** | ns |
| FE | -0.053 | 0.096 | 0.386 | -0.045 | **-0.439** | **-0.435** | -0.213 | -0.378 | -0.324 | -0.286 | 0.137 | 0.091 | 0.166 | 0.360 | **0.564** | 1.000 | ns |
| AL | **-0.552** | **-0.528** | 0.014 | 0.209 | -0.139 | -0.226 | -0.046 | **-0.440** | 0.115 | 0.197 | **0.463** | **0.558** | **0.486** | -0.179 | 0.335 | 0.211 | 1.000 |

*Sample size N=26; Values bellow the diagonal corresponds to the Person correlation coefficient (r), significant correlations are indicated in bold; Values above the diagonal correspond to the p-value of the significance levels for the correlations: ns – non-significant; * – significant at P-value < 0.05; ** – significant at P-value < 0.01; *** – significant at P-value < 0.001.*

*Quality traits’ abbreviations: PR – protein; FI – fiber; FT – fat; BD – breakdown; SB1 –setback1; FV – final viscosity; PV – peak viscosity; TV – trough viscosity;* b* *– yellow/blue index; TCC – total carotenoids; AT – α-tocopherol; DT – δ-tocopherol; GT – γ-tocopherol; PH – total free phenolic compounds; CU –* p*-coumaric acid; FE – ferulic acid; AL – volatile aldehydes*
