# Supplementary material for: Setting Up Decision-Making Tools toward a Quality-Oriented Participatory Maize Breeding Program
Source: Front Plant Sci. 2017 Dec 22;8:2203. doi: 10.3389/fpls.2017.02203 (PMC5744637; doi:10.3389/fpls.2017.02203)
Supplement: Supplementary file 6 [file Table6.docx]

***Supplementary Material***

**Setting up decision-making tools towards a quality-oriented participatory maize breeding program**

**Authors**

Mara Lisa Alves^1^, Cláudia Brites^2^, Manuel Paulo^2^, Bruna Carbas^3^, Maria Belo^1^, Pedro Mendes-Moreira^2^, Carla Brites^3^, Maria do Rosário Bronze^1, 4, 5^, Jerko Gunjača^6,7^, Zlatko Šatović^6,7^, Maria Carlota Vaz Patto^1^*

**Correspondence**

*Corresponding author: [cpatto@itqb.unl.pt](mailto:cpatto@itqb.unl.pt)

**Table S6.** Pearson correlation coefficients between quality traits and the first four principal components (PC) scores (PC1 to PC4), and the eigenvalues and percentage of variance for the four principal components.

| No | Trait | PC1 |  | PC2 |  | PC3 |  | PC4 |  |
| --- | --- | --- | --- | --- | --- | --- | --- | --- | --- |
| 1 | PR | -0.863 | *** | -0.300 | ns | 0.326 | ns | -0.095 | ns |
| 2 | FI | -0.907 | *** | -0.124 | ns | 0.222 | ns | -0.121 | ns |
| 3 | FT | -0.203 | ns | 0.580 | ** | -0.402 | * | -0.020 | ns |
| 4 | BD | 0.669 | *** | 0.107 | ns | -0.582 | ** | -0.018 | ns |
| 5 | SB1 | 0.147 | ns | -0.472 | * | -0.717 | *** | -0.155 | ns |
| 6 | *b** | 0.506 | ** | -0.688 | *** | 0.223 | ns | 0.029 | ns |
| 7 | TCC | 0.611 | *** | -0.626 | *** | 0.250 | ns | 0.014 | ns |
| 8 | AT | 0.872 | *** | 0.195 | ns | 0.039 | ns | -0.118 | ns |
| 9 | DT | 0.863 | *** | 0.128 | ns | 0.120 | ns | -0.176 | ns |
| 10 | GT | 0.468 | * | 0.139 | ns | 0.533 | ** | -0.553 | ** |
| 11 | PH | -0.281 | ns | 0.447 | * | -0.210 | ns | -0.718 | *** |
| 12 | CU | 0.120 | ns | 0.676 | *** | 0.132 | ns | 0.552 | ** |
| 13 | FE | -0.046 | ns | 0.765 | *** | 0.265 | ns | -0.045 | ns |
| 14 | AL | 0.624 | *** | 0.336 | ns | 0.289 | ns | 0.094 | ns |
|  | Eigenvalue | 4.915 |  | 2.969 |  | 1.796 |  | 1.231 |  |
|  | % of variance | 35.11 |  | 21.21 |  | 12.83 |  | 8.79 |  |

*P-value of the significance levels of correlations indicated as: ns – non-significant; * – significant at P < 0.05; ** – significant at P < 0.01; *** – significant at P < 0.001*

*Quality traits’ abbreviations: PR – protein; FI – fiber; FT – fat; BD – breakdown; SB1 –setback1;* b* *– yellow/blue index; TCC – total carotenoids; AT – α-tocopherol; DT – δ-tocopherol; GT – γ-tocopherol; PH – total free phenolic compounds; CU –* p*-coumaric acid; FE – ferulic acid; AL – volatile aldehydes*.
