# Supplementary material for: Setting Up Decision-Making Tools toward a Quality-Oriented Participatory Maize Breeding Program
Source: Front Plant Sci. 2017 Dec 22;8:2203. doi: 10.3389/fpls.2017.02203 (PMC5744637; doi:10.3389/fpls.2017.02203)
Supplement: Supplementary file 7 [file Table7.docx]

***Supplementary Material***

**Setting up decision-making tools towards a quality-oriented participatory maize breeding program**

**Authors**

Mara Lisa Alves^1^, Cláudia Brites^2^, Manuel Paulo^2^, Bruna Carbas^3^, Maria Belo^1^, Pedro Mendes-Moreira^2^, Carla Brites^3^, Maria do Rosário Bronze^1, 4, 5^, Jerko Gunjača^6,7^, Zlatko Šatović^6,7^, Maria Carlota Vaz Patto^1^*

**Correspondence**

*Corresponding author: [cpatto@itqb.unl.pt](mailto:cpatto@itqb.unl.pt)

Table S7. Within-population genetic diversity estimates in 26 maize populations (N = 780).

| Population | N | N_av_ | N_ar_ | N_pr_ | H_O_ | H_E_ | F_IS_ | P-value HWE |
| --- | --- | --- | --- | --- | --- | --- | --- | --- |
| Broa-048 | 30 | 3.2 | 3.1 | 1 | 0.502 | 0.468 | -0.073 | ns |
| Broa-057 | 30 | 3.4 | 3.3 | 0 | 0.462 | 0.477 | 0.032 | ns |
| Broa-065 | 30 | 3.5 | 3.4 | 1 | 0.487 | 0.507 | 0.041 | ns |
| Broa-070 | 30 | 3.2 | 3.1 | 0 | 0.487 | 0.504 | 0.033 | ns |
| Broa-092 | 30 | 3.3 | 3.2 | 0 | 0.458 | 0.482 | 0.049 | ns |
| Broa-102 | 30 | 3.6 | 3.5 | 0 | 0.466 | 0.490 | 0.049 | ns |
| Broa-113 | 30 | 3.6 | 3.5 | 0 | 0.566 | 0.549 | -0.032 | ns |
| Broa-136 | 30 | 3.3 | 3.2 | 0 | 0.514 | 0.525 | 0.020 | ns |
| Broa-142 | 30 | 3.3 | 3.2 | 1 | 0.474 | 0.535 | 0.113 | ** |
| Broa-148 | 30 | 3.0 | 2.9 | 0 | 0.488 | 0.483 | -0.010 | ns |
| Broa-164 | 30 | 3.1 | 3.0 | 0 | 0.491 | 0.463 | -0.059 | ns |
| Broa-172 | 30 | 3.4 | 3.3 | 1 | 0.506 | 0.508 | 0.004 | ns |
| Broa-186 | 30 | 3.2 | 3.1 | 2 | 0.490 | 0.487 | -0.007 | ns |
| Broa-214 | 30 | 3.0 | 2.9 | 0 | 0.446 | 0.445 | -0.003 | ns |
| Broa-CMSPH3 | 30 | 3.1 | 3.0 | 0 | 0.525 | 0.518 | -0.015 | ns |
| Broa-CMSPH8 | 30 | 2.8 | 2.8 | 1 | 0.424 | 0.405 | -0.048 | ns |
| Amiúdo | 30 | 4.0 | 3.8 | 1 | 0.503 | 0.526 | 0.042 | ns |
| Bastos | 30 | 3.5 | 3.4 | 0 | 0.503 | 0.530 | 0.052 | ns |
| Pigarro | 30 | 3.8 | 3.7 | 0 | 0.493 | 0.523 | 0.057 | ns |
| Verdeal da Aperrela | 30 | 3.5 | 3.4 | 1 | 0.468 | 0.516 | 0.093 | *** |
| Aljezur | 30 | 4.0 | 3.9 | 0 | 0.566 | 0.591 | 0.042 | ns |
| Castro Verde | 30 | 3.8 | 3.7 | 0 | 0.457 | 0.498 | 0.082 | ns |
| Estica | 30 | 4.1 | 4.0 | 0 | 0.549 | 0.588 | 0.065 | ns |
| Fisga | 30 | 3.9 | 3.8 | 1 | 0.536 | 0.560 | 0.043 | ns |
| Fandango | 30 | 3.7 | 3.6 | 0 | 0.551 | 0.563 | 0.022 | ns |
| BS22(R)C6 | 30 | 2.9 | 2.8 | 0 | 0.477 | 0.468 | -0.019 | ns |
| Average |  | 3.4 | 3.3 |  | 0.496 | 0.508 | 0.022 |  |

*N* – *sample size; N_av_* – *average number of alleles; N_ar_* – *average number of alleles per locus independent of sample size (allelic richness); N_pr_* – *total number of private alleles; H_O_* – *observed heterozygosity; H_E_* – *expected heterozygosity; F_IS_* – *inbreeding coefficient; P-value HWE: The probability global test for Hardy-Weinberg equilibrium (HWE) for each population was based on Markov chain method. ns* – *non-significant; *** – *significant at P < 0.01; **** – *significant at P < 0.001*
