# Supplementary material for: Setting Up Decision-Making Tools toward a Quality-Oriented Participatory Maize Breeding Program
Source: Front Plant Sci. 2017 Dec 22;8:2203. doi: 10.3389/fpls.2017.02203 (PMC5744637; doi:10.3389/fpls.2017.02203)
Supplement: Supplementary file 8 [file Table8.docx]

***Supplementary Material***

**Setting up decision-making tools towards a quality-oriented participatory maize breeding program**

**Authors**

Mara Lisa Alves^1^, Cláudia Brites^2^, Manuel Paulo^2^, Bruna Carbas^3^, Maria Belo^1^, Pedro Mendes-Moreira^2^, Carla Brites^3^, Maria do Rosário Bronze^1, 4, 5^, Jerko Gunjača^6,7^, Zlatko Šatović^6,7^, Maria Carlota Vaz Patto^1^*

**Correspondence**

*Corresponding author: [cpatto@itqb.unl.pt](mailto:cpatto@itqb.unl.pt)

Table S8. Pairwise F_ST_ values between farmers’ populations, pairwise F_ST_ values between participatory bred (PPB) populations, and pairwise F_ST_ values between all maize populations.

| Parameter | Farmers’ populations | | PPB populations | | All populations | |
| --- | --- | --- | --- | --- | --- | --- |
|  | *F_ST_* | Between populations | *F_ST_* | Between populations | *F_ST_* | Between populations |
| Average | 0.099 |  | 0.113 |  | 0.124 |  |
| Minimum | 0.030 | Broa-142 / Broa-172 | 0.005 | Estica / Fisga | 0.005 | Estica / Fisga |
| Maximum | 0.262 | Broa-214 / Broa-CMSPH8 | 0.233 | Verdeal da Aperrela / Castro Verde | 0.377 | Broa-CMSPH8 / BS22(R)C6 |

*F_ST_ stands for fixation index*
