# Supplementary material for: Setting Up Decision-Making Tools toward a Quality-Oriented Participatory Maize Breeding Program
Source: Front Plant Sci. 2017 Dec 22;8:2203. doi: 10.3389/fpls.2017.02203 (PMC5744637; doi:10.3389/fpls.2017.02203)
Supplement: Supplementary file 9 [file Table9.docx]

***Supplementary Material***

**Setting up decision-making tools towards a quality-oriented participatory maize breeding program**

**Authors**

Mara Lisa Alves^1^, Cláudia Brites^2^, Manuel Paulo^2^, Bruna Carbas^3^, Maria Belo^1^, Pedro Mendes-Moreira^2^, Carla Brites^3^, Maria do Rosário Bronze^1, 4, 5^, Jerko Gunjača^6,7^, Zlatko Šatović^6,7^, Maria Carlota Vaz Patto^1^*

**Correspondence**

*Corresponding author: [cpatto@itqb.unl.pt](mailto:cpatto@itqb.unl.pt)

**Table S9.** Average chord distance between farmers’ populations, average chord distance between participatory bred (PPB) populations, and overall Cavalli-Sforza–Edwards’ chord distances between all maize populations.

| Parameter | Farmers’ populations | | PPB populations | | All populations | |
| --- | --- | --- | --- | --- | --- | --- |
|  | *D_CSE_* | Between populations | *D_CSE_* | Between populations | *D_CSE_* | Between populations |
| Average | 0.078 |  | 0.096 |  | 0.104 |  |
| Minimum | 0.035 | Broa-136 / Broa-172 | 0.021 | Estica / Fisga | 0.021 | Estica / Fisga |
| Maximum | 0.183 | Broa-214 / Broa-CMSPH8 | 0.164 | Verdeal da Aperrela / Castro Verde | 0.281 | Broa-CMSPH8 / BS22(R)C6 |

*D_CSE_ - Pairwise Cavalli-Sforza–Edwards’ chord distance*
