# Supplementary material for: Setting Up Decision-Making Tools toward a Quality-Oriented Participatory Maize Breeding Program
Source: Front Plant Sci. 2017 Dec 22;8:2203. doi: 10.3389/fpls.2017.02203 (PMC5744637; doi:10.3389/fpls.2017.02203)
Supplement: Supplementary file 10 [file Image1.pdf]

Supplementary Material

Setting up decision-making tools towards a quality-oriented participatory maize breeding program

Authors

Mara Lisa Alves[1], Cláudia Brites[2], Manuel Paulo[2], Bruna Carbas[3], Maria Belo[1], Pedro Mendes-Moreira[2], Carla Brites[3], Maria do Rosário Bronze[1, 4, 5], Jerko Gunjača[6,7], Zlatko Šatović[6,7], Maria Carlota Vaz Patto[1]*

Correspondence

*Corresponding author: cpatto@itqb.unl.pt

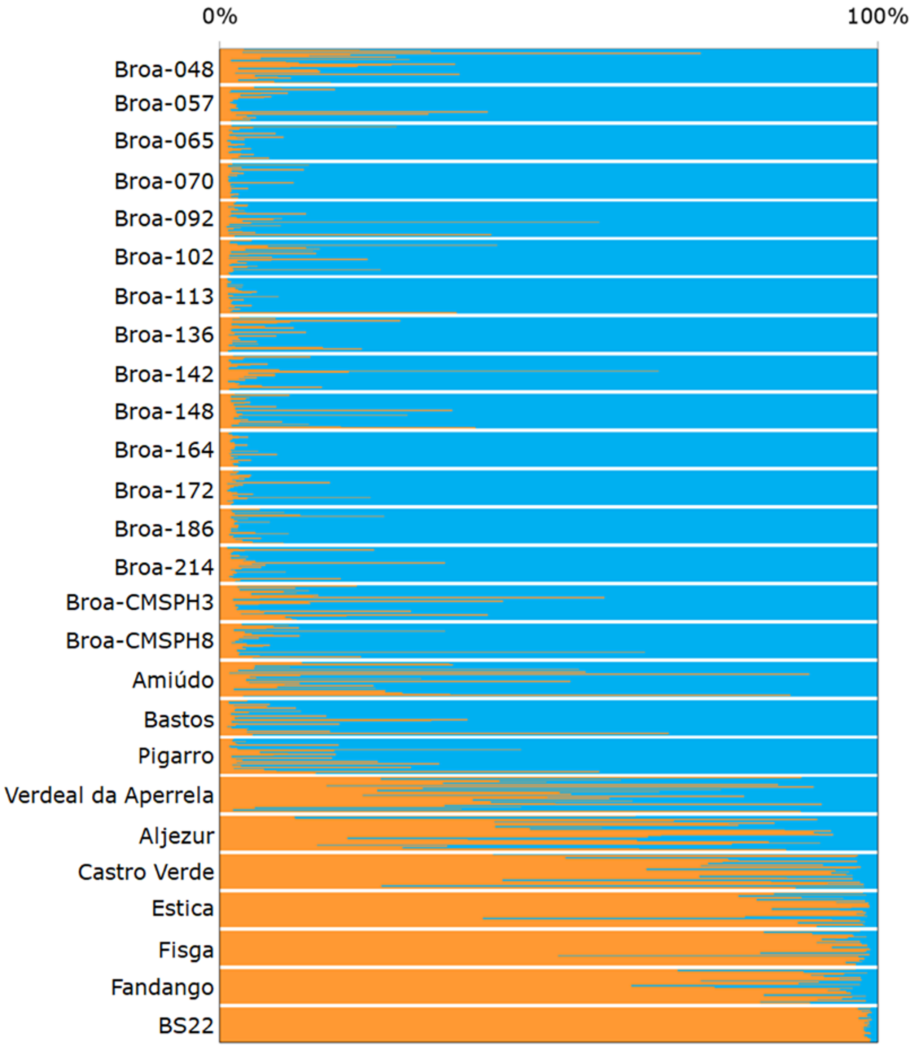

Figure S1. Proportion of membership of each maize population in each of the two gene pools inferred from multi-locus microsatellite data using a model-based clustering method. Each horizontal line within each population corresponds to an individual plant. Gene pool A is depicted in blue; gene pool B is depicted in orange. The *BS22(R)C6* synthetic population from the US, abbreviated for *BS22* in the figure.
